# Increased interleukin-26 in the peripheral joints of patients with axial spondyloarthritis and psoriatic arthritis, co-localizing with CD68-positive synoviocytes

**DOI:** 10.3389/fimmu.2024.1355824

**Published:** 2024-05-10

**Authors:** Ariane Hammitzsch, Andreas Ossadnik, Quirin Bachmann, Helga Merwald-Fraenk, Georg Lorenz, Matthias Witt, Franziska Wiesent, Heinrich Mühlhofer, Davide Simone, Paul Bowness, Uwe Heemann, Martin Arbogast, Philipp Moog, Christoph Schmaderer

**Affiliations:** ^1^Department of Nephrology, Klinikum Rechts der Isar, School of Medicine, Technical University of Munich, Munich, Germany; ^2^Amedes Holding AG, Ambulatory Healthcare Center (MVZ) Endokrinologikum München, Munich, Germany; ^3^Department of Nephrology and Rheumatology, Klinik Augustinum München, Munich, Germany; ^4^Rheumazentrum Bad Abling Erding, Erding, Germany; ^5^Clinic and Policlinic of Orthopaedics and Sports’ Orthopaedics, Klinikum Rechts der Isar, School of Medicine, Technical University of Munich, Munich, Germany; ^6^Kennedy Institute of Rheumatology, University of Oxford, Oxford, United Kingdom; ^7^Botnar Research Centre, Nuffield Department of Orthopaedics, Rheumatology and Musculoskeletal Sciences, Medical Sciences Division, University of Oxford, Oxford, United Kingdom; ^8^Department of Rheumatic Orthopedics and Hand Surgery, Klinik Oberammergau, Waldburg-Zeil Kliniken GmbH und Co KG, Oberammergau, Germany

**Keywords:** *IL26*, Th17 cells, synvovial tissue, axial spondyloarthritis, psoriatic arthritis

## Abstract

**Objectives:**

*IL26* levels are elevated in the blood and synovial fluid of patients with inflammatory arthritis. *IL26* can be produced by Th17 cells and locally within joints by tissue-resident cells. *IL26* induces osteoblast mineralization *in vitro*. As osteoproliferation and Th17 cells are important factors in the pathogenesis of axial spondyloarthritis (axSpA), we aimed to clarify the cellular sources of *IL26* in spondyloarthritis.

**Methods:**

Serum, peripheral blood mononuclear cells (*n* = 15–35) and synovial tissue (*n* = 3–9) of adult patients with axSpA, psoriatic arthritis (PsA) and rheumatoid arthritis (RA) and healthy controls (HCs, *n* = 5) were evaluated by ELISA, flow cytometry including PrimeFlow assay, immunohistochemistry and immunofluorescence and quantitative PCR.

**Results:**

Synovial tissue of axSpA patients shows significantly more *IL26*-positive cells than that of HCs (*p* < 0.01), but numbers are also elevated in PsA and RA patients. Immunofluorescence shows co-localization of *IL26* with CD68, but not with CD3, SMA, CD163, cadherin-11, or CD90. *IL26* is elevated in the serum of RA and PsA (but not axSpA) patients compared with HCs (*p* < 0.001 and *p* < 0.01). However, peripheral blood CD4^+^ T cells from axSpA and PsA patients show higher positivity for *IL26* in the PrimeFlow assay compared with HCs. CD4^+^ memory T cells from axSpA patients produce more *IL26* under Th17-favoring conditions (IL-1β and IL-23) than cells from PsA and RA patients or HCs.

**Conclusion:**

*IL26* production is increased in the synovial tissue of SpA and can be localized to CD68^+^ macrophage-like synoviocytes, whereas circulating *IL26*^+^ Th17 cells are only modestly enriched. Considering the osteoproliferative properties of *IL26*, this offers new therapeutic options independent of Th17 pathways.

## Introduction

Axial spondyloarthritis (axSpA) and psoriatic arthritis (PsA) belong to a group of immune-mediated inflammatory diseases with primarily axial but also peripheral arthritis, with a prevalence of approximately 0.54% in Europe (1, 2). These diseases additionally present with extra-articular manifestations like uveitis, inflammatory bowel disease and psoriasis. As the onset of disease predominates in the third decade, socioeconomic and personal effects are high and long-lasting, demanding efficient therapy. The common denominators of axSpA and PsA are inflammation of the entheses and osteoproliferation along the tendons and capsules. Presumably, inflammation precedes new bone formation and early halting of inflammation by therapies like non-steroidal anti-inflammatory drugs (NSAIDs), anti-TNF, anti-IL-17A/F and Janus kinase inhibition (JAKi) can avoid osteoproliferation and ultimately ankylosis. However, the precise mechanisms and players of this osteoproliferation have not been satisfactorily elucidated yet.

The efficacy of anti-IL-17A/F treatment in clinical studies together with genetic and functional evidence underlines the role of the IL-23/IL-17A pathway in the pathogenesis of SpA (3–5). Main players of the IL-23/IL-17A pathway in SpA are “pathogenic” Th17 cells, producing IL-17A, IL-17F, IL-22, GM-CSF and IFNγ (6). Pathogenic Th17 cells arise from Th17 cells, which are responsible for the maintenance of tissue homeostasis at mucosal barriers and host defense against fungi and extracellular bacteria, in the presence of IL-23 (7–9). *IL26*, a member of the IL-10 family, is also produced by pathogenic Th17 cells (10, 11). It uniquely combines proinflammatory with antimicrobial, DNA-binding and DNA-shuttling properties (12, 13). Elevated systemic levels and locally increased expression of *IL26* have been reported in chronic inflammatory diseases like Crohn’s disease, rheumatoid arthritis, SpA, psoriasis and ANCA-associated vasculitis (11, 13–16). *IL26* signaling is mediated by multiple pathways, including a classical cytokine receptor (IL-10R2 and IL-20R1) leading to activation of JAK1 and TYK2, and direct cell membrane penetration. *IL26* expression has also been reported in CD8^+^ T cells, mucosal-associated invariant T (MAIT) cells, NKp44^+^ NK cells, γδT cells, innate lymphoid cells type 3 (ILC3), CD68^+^ macrophage-like and synoviolin^+^ fibroblast-like synoviocytes and synovial myofibroblasts (15–21). Effects have been observed on human intestinal and bronchial epithelial cells, endothelial cells, fibroblasts, monocytes, NK and B cells, plasmacytoid dendritic cells and neutrophils (12, 15, 16, 22–28).

*IL26* also increases mineralization in human osteoblasts, and inhibits receptor activator of nuclear factor κB ligand-induced osteoclastogenesis and bone-resorbing activity of mature osteoclasts, making it an interesting therapeutic target for preventing osteoproliferation (16, 24). Additionally, the capacity of *IL26* to bind DNA released during inflammation-induced tissue damage renders *IL26* a key driver of chronic inflammation. This provides another interesting feature of anti-*IL26* therapy for axSpA and PsA because, with current therapeutic options (NSAIDs, anti-TNF, anti-IL-17A/F, and JAKi), approximately 40% of patients will not enter sustained remission. Therefore, the need for new therapies remains high in these patients.

In this study we comparatively investigate the expression and cellular sources of *IL26* in the peripheral blood and peripheral synovial tissue of anti-TNF therapy-naive patients with axSpA, PsA, and RA. We demonstrate significantly increased local expression of *IL26* in axSpA, potentially implicating a significant role of *IL26* in osteoproliferation and a new therapeutic target.

## Patients and methods

### Patient samples

Venous blood (max. 50 ml) and synovial tissue were obtained from patients with axSpA (*n* = 29/9, modified New York criteria), PsA [*n* = 20/3, Classification Criteria for Psoriatic Arthritis (CASPAR)], and RA (*n* = 15/7, American College of Rheumatology/European League against Rheumatism 2010 criteria) and from healthy controls (HCs, *n* = 35/5), after obtaining informed written consent, with ethical permission (100/18 S, Ethical Committee of the Faculty of Medicine, Technical University Munich and 06/Q1606/139 Oxford University Hospitals NHS Foundation Trust). The study has been registered within the German Clinical Trials Register (DRKS00014672).

Healthy donors were sex- and age-matched to the axSpA group. All research was performed in accordance with the relevant guidelines and regulations including the Helsinki Declaration.

Serum was isolated from a 4.7-ml serum tube (Sarstedt, Nümbrecht, Germany), aliquoted and stored at −80°C until analysis.

### Cell purification and cell culture

Peripheral blood mononuclear cells (PBMCs) were isolated by Ficoll density-gradient centrifugation (4 × 8 ml, CPT tube, BD, Franklin Lakes, USA). CD4^+^ T cells were then negatively, and CD14^+^ cells positively selected via magnetic beads (BioLegend, San Diego, USA, >90.96% purity on average), and cultured under stimulatory conditions as described in the online **Supplementary Material and Methods**. Cells were cryopreserved in FCS with 10% DMSO (Sigma-Aldrich, Burlington, USA) in liquid nitrogen until further analysis.

### Synovial tissue acquisition and preparation

Synovial tissue was obtained during orthopedic surgery or arthroscopy and processed as described in the online **Supplementary Materials and Methods**.

### ELISA

Serum levels of *IL26* were analyzed with a modified *IL26* ELISA kit (Elabscience, Wuhan, China). For detailed modifications, see the online **Supplementary Materials and Methods**.

### Immunohistochemistry

Tissue sections were prepared and stained with the DAKO Envision+ System HRP Kit (Dako, Santa Clara, USA), and images were acquired and quantitatively analyzed as described in the online **Supplementary Material and Methods**.

### Immunofluorescence

Immunofluorescence was performed according to an adapted protocol from Dakin S et al. with the incorporation of the Tyramide SuperBoost Kit (Invitrogen, Waltham, USA) (29). For detailed information, see the online **Supplementary Material and Methods** and **Supplementary Table S1**.

### Flow cytometry and PrimeFlow assay

PBMCs were stimulated and stained for surface phenotyping of IL-1R1 and chemokine receptors and detection of *IL26*-positive cells in combination with surface markers, and intracellular cytokines as described in the online **Supplementary Materials and Methods** and **Supplementary Tables S2**, **S3**.

### qPCR

qPCR was performed on CD4^+^ T cells (1 × 10^6^) after 6 days of *in vitro* culture as further described in the online **Supplementary Materials and Methods** and **Supplementary Table S4**.

### Statistical analysis

GraphPad Prism software version 5 was used for statistical analysis. Statistical tests were applied as stated in the figure legends. Significance was defined as *p* ≤ 0.05.

## Results

### Serum levels of *IL26* are significantly elevated in PsA and RA, but not in axSpA

**Figure 1:**
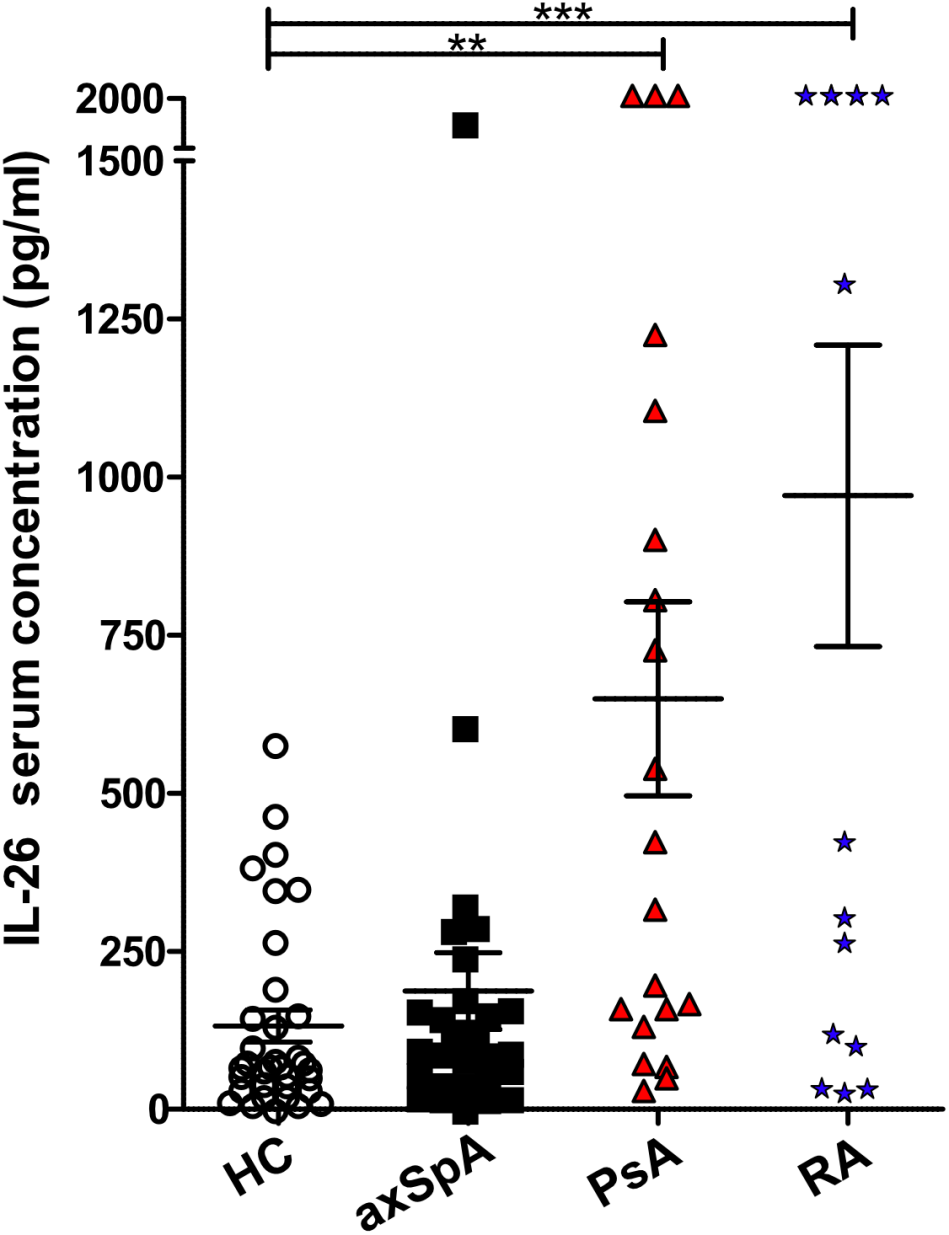
Increased IL-26 levels in the serum of psoriatic arthritis (PsA) and rheumatoid arthritis (RA) patients. IL-26 serum levels were quantified by ELISA in 35 HCs (white), 29 axial spondyloarthritis (axSpA) (black), 20 PsA (red) and 15 RA (blue) patients. Preincubation with heat-aggregated bovine IgG and signal amplification with biotinyl-tyramide were applied in order to minimize false results through interfering antibodies (30). Statistical analysis: mean ± SEM, **p < 0.01, ***p < 0.001 (one-way ANOVA followed by Dunnett post-test for multiple comparisons).

Two studies have reported elevated levels of *IL26* in the serum and synovial fluid of RA and SpA patients (15, 16). However, SpA patients were not further classified, the numbers were low (*n* = 9–15) and treatment was either unclear or included anti-TNFα. Therefore, we compared the serum levels of *IL26* from anti-TNF-naive patients with axSpA, PsA and RA with age- and sex-matched HCs. Detailed patient characteristics are shown in **Table 1**. Serum levels of *IL26* were not significantly elevated in axSpA patients compared with HCs (**Figure 1**, mean 187.2 pg/ml, SEM 60.78 pg/ml and mean 131.8 pg/ml, SEM 25.19 pg/ml). However, significantly increased serum levels of *IL26* were evident in patients with PsA and RA (mean 649.3 pg/ml, SEM 153.2 pg/ml and mean 970.5 pg/ml, SEM 238.2 pg/ml; *p* < 0.01 and *p* < 0.001). Corresponding synovial fluid for comparative analysis was not available. Correlation analysis of *IL26* serum levels with markers of disease activity and inflammation revealed a weak, yet significant positive correlation only with CRP levels in axSpA patients (**Supplementary Figure S1**, *r* = 0.467, 95% CI [*r*] = 0.088 to 0.728, *r*^2 =^ 0.218, *p* < 0.05).

**Table 1:** Study population characteristics—blood samples.

	HCs (*n* = 35)	axSpA (*n* = 29)	PsA (*n* = 20)	RA (*n* = 15)
**Male/female, *n***	17/18	16/13	13/7	1/14
**Age, years (median and max–min)**	35.0 (67/20)	34.0 (66/22)	52.5 (77/28)	57.0 (79/31)
**HLA-B27 positive, %**	na	82.8	15.0^a^	na
**Rheumatoid factor positive, %**	na	na	na	66.7
**Anti-CCP positive, %**	na	na	na	60.0^b^
**BASDAI (mean, SD)**	na	4.0 (1.8)^c^	na	na
**BASFI (mean, SD)**	na	1.8 (1.7)^c^	na	na
**BASMI (mean, SD)**	na	2.1 (1.9)^c^	na	na
**DAS28 CRP (mean, SD)**	na	na	2.7 (0.7)^d^	4.0 (1.1)^d^
**CRP, mg/l (mean, SD)**	na	6.8 (9.3)^e^	9.0 (8.5)^e^	10.2 (11.2)^e^
**BSG, mm/1 h (mean, SD)**	na	19.4 (18.1)^f^	13.9 (11.6)^f^	16.6 (9.2)^f^
Treatment				
Anti-TNF, *n*	na	0/29	0/20	0/15
DMARD, *n*	na	4/29	8/20	8/15
Steroids, *n*	na	0/29	0/20	0/15
No treatment, *n*	na	24/29	12/20	7/15
Comorbidities				
Uveitis, *n*	0/35	3/29	1/20	1/15
Psoriasis, *n*	0/35	1/29	11/20	0/15
IBD, *n*	0/35	3/29	0/20	0/15

HLA-B27, human leucocyte antigen-B27; na, not applicable; RF, rheumatoid factor; anti-CCP, anti-cyclic citrullinated peptide; BASDAI, Bath Ankylosing Spondylitis Disease Activity Index; BASFI, Bath Ankylosing Spondylitis Functional Index; BASMI, Bath Ankylosing Spondylitis Metrology Index; DAS28 CRP, Disease Activity Score 28 CRP; CRP, C-reactive protein; DMARD, disease-modifying antirheumatic drug; IBD, inflammatory bowel disease.

a Data available for 13 PsA patients.

b Data available for 14 RA patients.

c Data available for 26 axSpA patients.

d Data available for 11 PsA and 10 RA patients.

e Data available for 25 axSpA, 16 PsA and 14 RA patients.

f Data available for 24 axSpA, 13 PsA and 8 RA patients.

### No significant enrichment of *IL26*-producing Th17 cells in the peripheral blood of axSpA and PsA patients

**Figure 2:**
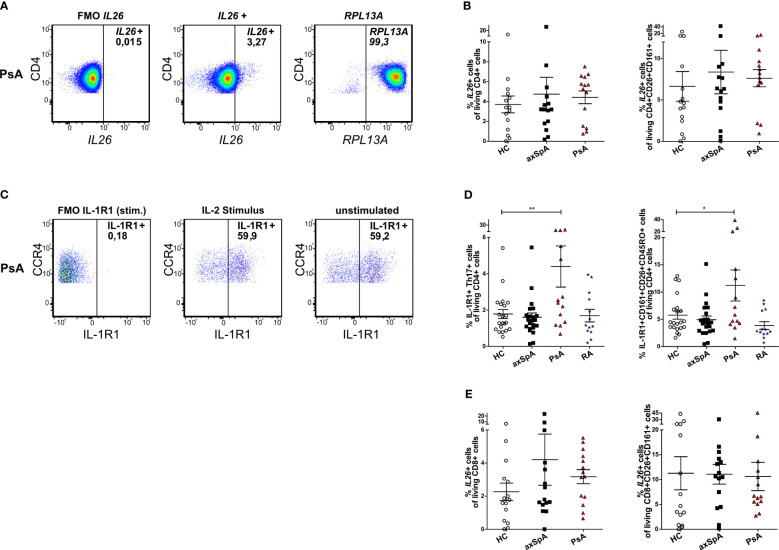
*IL26*-expressing cells are only slightly increased in circulating CD4^+^ and CD8^+^ T cells from patients with axSpA and PsA. **(A)** Representative flow cytometry plots of total *IL26*^+^CD4^+^ T cells with the PrimeFlow assay in a PsA patient. RPLA13A represents the positive control for the assay. **(B)** Percentages of total *IL26*^+^CD4^+^ T cells (left) and percentages of *IL26*^+^ cells of CD26^+^CD161^+^CD4^+^ T cells (right) for HCs (*n* = 15; white) and axSpA (*n* = 15; black) and PsA (*n* = 14; red) patients gated from PBMCs stimulated *in vitro* with PMA and ionomycin in the PrimeFlow assay. **(C)** Representative flow cytometry plots of total IL-1R1^+^ Th17 cells in a PsA patient comparing IL-2 stimulation to unstimulated conditions. **(D)** Percentages of IL-1R1^+^ Th17 cells (Th17 cells gated as CD4^+^CD45RO^+^CD161^+^CCR6^+^CCR4^+^CXCR3^−^) of CD4^+^ cells from HCs (*n* = 21; white) and axSpA (*n* = 22; black), PsA (*n* = 14; red), and RA (*n* = 13; blue) patients after *in vitro* stimulation with IL-2 (1,000 IU/ml) overnight (left) and percentages of IL-1R1^+^CD26^+^CD161^+^ cells of CD4^+^ cells (right). **(E)** Percentages of total *IL26*^+^CD8^+^ T cells (left) and percentages of *IL26*^+^ cells of CD26^+^CD161^+^CD8^+^ T cells (right) for HCs (*n* = 15; white) and axSpA (*n* = 15; black) and PsA (*n* = 14; red) patients gated from PBMCs stimulated *in vitro* with PMA and ionomycin. Statistical analysis: mean ± SEM, *p < 0.05, **p < 0.01 (one-way ANOVA followed by Dunnett post-test for multiple comparisons).

As Th17 cells reportedly are the main producers of *IL26* in the peripheral blood of HCs (12), we analyzed the cellular sources of *IL26* in the peripheral blood by flow cytometry. Because of a lack of a reliable anti-*IL26* antibody for flow cytometry, we analyzed Th17 cells from the peripheral blood of axSpA and PsA patients and HCs for *IL26* expression with the PrimeFlow assay (*in-situ* hybridization utilizing branched DNA technology with target-specific probes compatible with conventional flow cytometry). Total PBMCs were stimulated for 4 h *in vitro* with PMA and ionomycin prior to analysis. **Figures 2A, B** illustrate higher, yet not significantly higher percentages of **IL26*^+^* cells in total CD4^+^ T cells in axSpA (mean 4.74%, SEM 1.70%) and PsA (mean 4.42%, SEM 0.64%) patients compared with HCs (mean 3.70%, SEM 0.85%; ns). The percentages of **IL26*^+^* cells of CD4^+^CD26^+^CD161^+^ cells, a surface pattern known to characterize CD4^+^ T cells that harbor the majority of IL-17A-producing cells, are also slightly increased in axSpA (mean 8.36%, SEM 2.63%) and PsA (mean 7.62%, SEM 1.03%) patients compared with HCs (mean 6.63%, SEM 1.80%; ns) (31). For the gating strategy, see **Supplementary Figure S2A**.

As these experiments showed only a trend for higher percentages of *IL26*^+^ Th17 cells in axSpA and PsA, an alternative strategy for the identification of *IL26*-producing Th17 cells was applied using surface staining for IL-1R1 as a surrogate marker in an extended set of patient samples including approximately half of the PsA samples and one-third of the axSpA and HC samples from the PrimeFlow assay. *IL26* secretion is restricted to IL-1R1^+^ Th17 cells and can be elicited by IL-1β or TCR activation (32). Th17 cells from axSpA, PsA and RA patients and HCs were characterized as CD4^+^CD45RO^+^CD161^+^CCR6^+^CCR4^+^CXCR3^−^ cells and analyzed for IL-1R1 (**Figures 2C, D**; for the gating strategy, see **Supplementary Figure S2B**). Percentages of these IL-1R1^+^ Th17 cells were only significantly increased in PsA patients (mean 4.40%, SEM 1.13%) compared with HCs (mean 1.78%, SEM 0.25%; *p* < 0.01, **Figure 2D**), but not in axSpA (mean 1.60%, SEM 0.23%) and RA (mean 1.69%, SEM 0.35%) patients. This finding was confirmed when analyzing the percentages of IL-1R1^+^CD161^+^CD26^+^CD45RO^+^ cells of living CD4^+^ cells in the same set of experiments (PsA: mean 11.20%, SEM 2.84% vs. HCs: mean 5.76%, SEM 0.74%; *p* < 0.05, **Figure 2D**) with no differences for axSpA (mean 4.93%, SEM 0.68%) and RA (mean 3.86%, SEM 0.71%).

As CD8^+^ T cells are another source of IL-17A, mainly by Tc17 (approximately 90%) and MAIT cells (approximately 4%), and are enriched in the synovial fluid from PsA and other SpA patients, where Tc17 cells show increased expression of *IL26*, we further investigated this cell type (21). Percentages of *IL26*^+^ cells in total CD8^+^ T cells tended to be higher in patients with axSpA (mean 4.21%, SEM 1.55%) and PsA (mean 3.17%, SEM 0.42%) compared with HCs (mean 2.26%, SEM 0.53%; ns, **Figure 2E** left). This increase could not be observed for percentages of *IL26*^+^ cells in CD8^+^CD26^+^CD161^+^ cells, a surface antigen pattern harboring most of IL-17A-producing CD8^+^ T cells (HCs: mean 11.29%, SEM 3.33% vs. axSPA: mean 11.09, SEM 1.99% and PsA: mean 10.63, SEM 2.85; ns; **Figure 2E** right) (21).

Taken together, these results do not show a clear enrichment of **IL26*^+^* Th17 and Tc17 cells in the circulation of patients with axSpA and PsA compared with HCs in our cohort of patients with moderate disease activity.

### CD4^+^ T cells from axSpA and PsA are primed for *IL26* production by IL-1β and IL-23 *in vitro*

**Figure 3:**
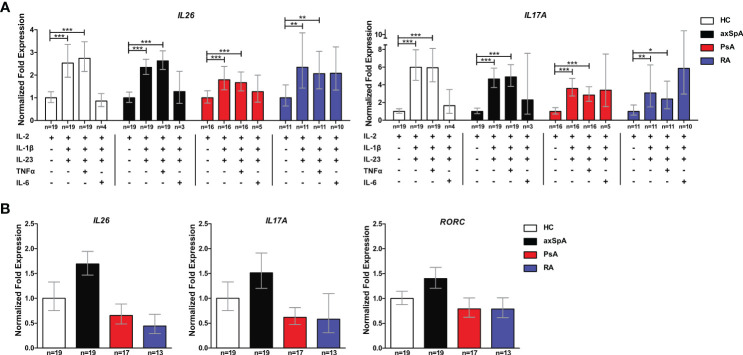
CD4^+^ T cells from axSpA patients are prone to *IL26* expression under typical Th17-favoring conditions *in vitro*. **(A)** Total CD4^+^ T cells from HCs (*n* = 20; white) and axSpA (*n* = 19; black), PsA (*n* = 18; red), and RA *n* = 13; blue) patients were incubated with different Th17-favoring cytokine combinations *in vitro* for 6 days, and the expression of *IL26* and *IL17A* was analyzed by qPCR in triplicates. Expression was normalized to two control genes (*ACT* and *RPL13A*) and is shown as fold expression in relation to basal stimulation with IL-2 only. **(B)** CD4^+^ T cells from the same cohort as in **(A)** were stimulated with IL-2, IL-1β, and IL-23 for 6 days *in vitro*, and fold expression (triplicates each) of the respective genes is shown in relation to HCs after normalizations for *ACT* and *RLP13A*. Statistical analysis: mean ± SEM, *p < 0.05, **p < 0.01, ***p < 0.001 [**(A)** repeated-measures ANOVA and **(B)** one-way ANOVA followed by Dunnett post-test for multiple comparisons].

Inflammation- and Th17 immunity-promoting conditions are enriched in joint tissue and are likely to increase *IL26* expression. Therefore, we investigated the capacity of CD4^+^ T cells from axSpA and PsA patients to express *IL26* at the mRNA level. *Ex-vivo* expression of *IL26* was detectable in higher percentages of axSpA (63.1%) and PsA (77.8%) CD4^+^ T cells compared with HCs (45%; ns; **Supplementary Figure S3A**). In order to evaluate if CD4^+^ T cells could be prompted to express *IL26* under Th17-favoring conditions, we incubated them for 6 days with different cytokine combinations of IL-2, IL-1β, IL-23, IL-6, and TNFα. After 6 days, levels of *IL26* expression were highest with a combination of classical Th17-favoring cytokines IL-1β and IL-23 relative to basal stimulation with IL-2 only (**Figure 3A**; HCs, axSpA, PsA: *p* < 0.001; RA: *p* < 0.01). Addition of TNFα had no additive effect compared with the combination of IL-2, IL-1β and IL-23, but remained significant compared with IL-2 (HCs, axSpA, PsA: *p* < 0.001; RA: *p* < 0.01). The inductive effect of IL-1β and IL-23 could also be observed for Th17-type cytokines like *IL17A*, *IL22*, and *IFNG*, but not for *IL4* and *IL1R1* (**Figure 3A**, **Supplementary Figure S3C**; *p* < 0.05 to *p* < 0.001). *RORC* expression was only significantly induced in PsA by the addition of IL-1β and IL-23 (**Supplementary Figure S3C**; *p* < 0.05). Comparing the inductive effect of the combination of IL-2, IL-1β, and IL-23 between the disease groups and HCs, we found a trend toward a higher relative expression in axSpA for *IL26*, *IL17A*, and *RORC* (**Figure 3B**). *IL22*, *IFNG*, and *IL1R1* expression levels were not different between the groups, but the relative expression of *IL4* was significantly increased in RA (**Supplementary Figure S3B**; *p* < 0.05).

These data demonstrate that CD4^+^ T cells from patients with axSpA are primed to express *IL26* upon encountering IL-1β and IL-23, classical cytokines for the induction of pathogenic Th17 cells.

### *IL26* expression is highest in the peripheral joint tissue of axSpA and co-localizes with CD68^+^ synoviocytes

**Figure 4:**
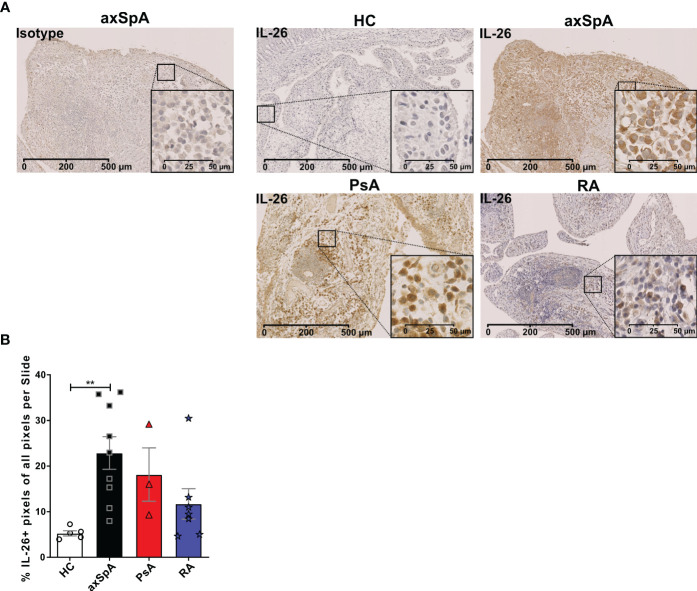
IL-26 expression in peripheral joints is increased in axSpA. **(A)** Representative slides of IL-26 expression by immunohistochemistry in peripheral synovial tissue from HCs (*n* = 5; white) and axSpA (*n* = 9; black), PsA (*n* = 3; red), and RA (*n* = 7; blue) patients. Isotype staining in 3,3′diaminobenzidine (brown) is shown on the far left. 20× (scale bar 500 μm) and inlay 80× (scale bar 50 μm). **(B)** Semiquantitative analysis of IL-26^+^ pixels in peripheral joint tissue sections from the cohort described in **(A)**. Statistical analysis **(B)**: mean ± SEM, **p < 0.01 (Kruskal–Wallis test).

**Figure 5:**
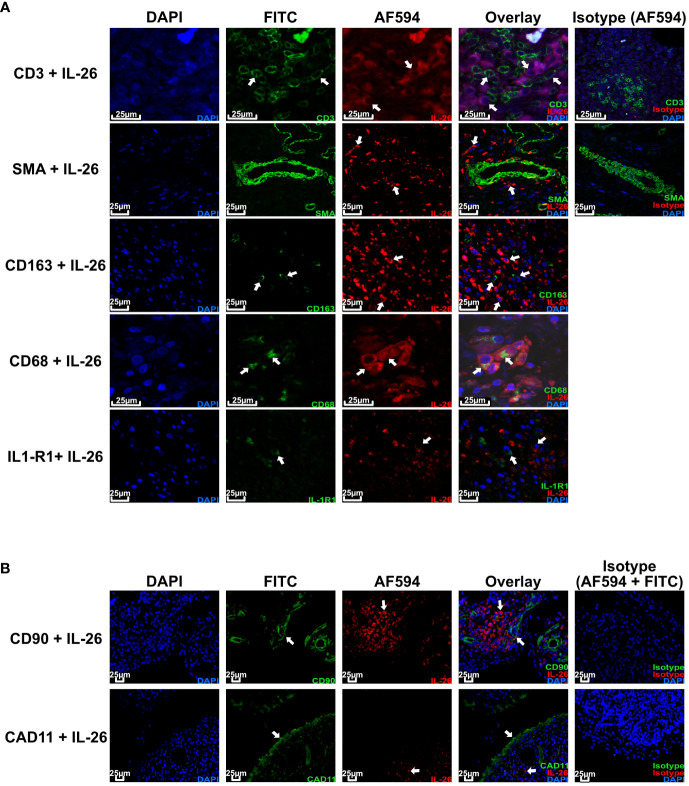
IL-26 co-localizes with CD68^+^ macrophage-like synoviocytes in axSpA. Representative confocal **(A)** and immunofluorescence images **(B)** of peripheral joint tissue from axSpA stained for IL-26 (red) and CD3/SMA/CD163/CD68/IL-1R1/Cadherin-11/CD90 (green). Blue shows DAPI nuclear stain. Scale bar 25 µm. Arrows indicate cells of interest for co-localization of the respective cell surface markers and IL-26.

Given the importance of local actions of *IL26* (high expression in RA joint tissue and synovial fluid of inflammatory arthritis), we deemed it of particular importance to examine the local production of *IL26* within the peripheral synovial tissue (15, 16). We performed comparative immunohistochemistry for *IL26* in peripheral synovial tissue from patients with axSpA, PsA and RA, and HCs. For detailed patient characteristics, see **Table 2**. Immunohistochemistry revealed significantly elevated expression of *IL26* in the lining and sublining layers of axSpA tissue compared with HCs by automated quantification (open source tool “IHC profiler,” **Figures 4A, B**; mean 22.88%, SEM 3.58% vs. mean 5.25%, SEM 0.58%; *p* < 0.01) (33). However, *IL26* expression was also increased in tissue from PsA and RA patients (mean 18.14%, SEM 5.84% and mean 11.71%, SEM 3.33%; ns). In order to further characterize the *IL26*-expressing cells in SpA tissue, immunofluorescence was performed. In axSpA, co-localization of *IL26* could only be shown with CD68, a marker for macrophage-like synoviocytes (**Figure 5A**, panel 4: CD68+*IL26*), but not for either the pan-T-cell marker CD3, the alternative macrophage-like synoviocyte marker CD163, the myofibroblast marker SMA, or IL-1R1, a marker of *IL26*-producing CD4^+^ T cells. The same staining pattern was apparent in PsA tissue (**Supplementary Figure S4**). *IL26* did also not co-localize with either cadherin-11, a marker of lining fibroblast-like synoviocytes, or CD90, a marker of sublining fibroblast-like synoviocytes (**Figure 5B**).

**Table 2:** Study population characteristics—synovial tissue samples.

	HCs (*n* = 5)	axSpA (*n* = 9)	PsA (*n* = 3)	RA (*n* = 7)
**Male/female, *n***	3/2	5/4	1/2	0/7
**Age, years (median and max–min)**	39 (65/20)	51 (71/35)	47 (73/22)	70 (77/53)
Joint (tissue origin)				
Hip, *n*	3	5^a^	0^a^	0^a^
Knee, *n*	2	2^a^	2^a^	5^a^
Shoulder, *n*	0	1^a^	0^a^	0^a^
Hand, *n*	0	0^a^	0^a^	1^a^
Reason for intervention				
Arthritis, *n*	0	9	3	7
FAI cam type, *n*	3	0	0	0
Arthrosis, *n*	2	0	0	0
**HLA-B27 positive, %**	na	77.78^b^	na	na
**Rheumatoid factor positive, %**	na	na	na	42.86^c^
**Anti-CCP positive, %**	na	na	na	42.86^d^
**BASDAI (mean, SD)**	na	5.05 (1.49)^e^	na	na
**BASFI (mean, SD)**	na	5.00 (2.26)^f^	1.50 (0)^f^	na
**BASMI (mean, SD)**	na	2.80 (0)^g^	na	na
**DAS28 CRP (mean, SD)**	na	na	3.66 (0)^f^	na
**CRP, mg/l (mean, SD)**	na	52.26 (95.19)^e^	46.94 (33.82)^h^	29.46 (50.78)^i^
**BSG, mm/1 h (mean, SD)**	na	35.0 (0)^g^	3.50 (2.12)^j^	14.75 (10.34)^k^
Treatment				
Anti-TNF, *n*	na	0/9	0/3	0/7
DMARD, *n*	na	1/9	1/3	3/7
Steroids, *n*	na	0/9	0/3	0/7
No treatment, *n*	na	8/9	2/3	4/7
Comorbidities				
Uveitis, *n*	0/5	0/9	0/3	0/7
Psoriasis, *n*	0/5	2/9	1/3	0/7
IBD, *n*	0/5	0/9	1/3	0/7

FAI, femoroacetabular impingement; cam, camshaft; HLA-B27, human leucocyte antigen-B27; na, not applicable; RF, rheumatoid factor; anti-CCP, anti-cyclic citrullinated peptide; BASDAI, Bath Ankylosing Spondylitis Disease Activity Index; BASFI, Bath Ankylosing Spondylitis Functional Index; BASMI, Bath Ankylosing Spondylitis Metrology Index; DAS28 CRP, Disease Activity Score 28 CRP; CRP, C-reactive protein; DMARD, disease-modifying antirheumatic drug; IBD, inflammatory bowel disease.

a Data available for eight axSPA, two PsA, and six RA patients.

b Data available for eight PsA patients.

c Data available for six RA patients.

d Data available for three RA patients.

e Data available for four axSpA patients.

f Data available for two axSpA patients and one PsA patient.

g Data available for one axSpA patient.

h Data available for three PsA patients.

i Data available for five RA patients.

j Data available for two PsA patients.

k Data available for four RA patients.

This finding confirms the local action of *IL26* in axSpA and PsA in the context of CD68^+^ macrophage-like synoviocytes, and not CD3^+^ T cells or fibroblast-like synoviocytes.

## Discussion

This is so far the largest comparative study of *IL26* in inflammatory arthritis with a focus on axSpA and PsA, clearly showing the increased expression of *IL26* in peripheral joints.

Our data uniquely add to previous findings of increased *IL26* expression in peripheral joints of axSpA by demonstrating *IL26* expression in axSpA as well as in PsA peripheral synovial tissue by immunohistochemistry and immunofluorescence (16). In our hands, *IL26* co-localizes with CD68^+^ macrophage-like synoviocytes in the sublining of the synovium. This is of special interest as efficacy of 12 weeks of secukinumab (ACR20 response rate of 65%) in peripheral SpA was accompanied by a significant reduction of synovial sublining CD68^+^ macrophages and CD15^+^ neutrophils (34). Compared with RA, state-of-the-art analysis of synovial tissue-resident macrophages (STMs) is not available from SpA yet. In human RA joint tissue, two populations of STMs are present: MerTK^−^CD206^−^ STMs that induce proinflammatory responses in synovial fibroblasts and MerTK^+^CD206^+^ STMs that induce resolution of inflammation and repair mechanisms (35). These MerTK^+^CD206^+^ STMs are predominant in RA in remission, and a proportion of less than 47.5% is an independent predictor for disease flares after tapering or discontinuation of treatment. It remains to be elucidated if *IL26*-positive CD68^+^ synovial macrophages stem from circulating monocytes or from synovial tissue-resident macrophages, which both have been demonstrated in the K/BxN serum-transfer arthritis model (36). An *ex vivo* study on synovial fluid from PsA showed an expansion of intermediate monocytes and macrophages (37). These cells demonstrated expression of *MRC1*, the gene encoding CD206, which was shown to be an important marker for STMs in RA. At the moment, we cannot exclude that the *IL26*^+^ synovial myeloid cells are not producing *IL26* themselves, but rather have shuttled extracellular DNA from cell death due to excessive inflammation in complex with *IL26* into their cytosol (13). Possible local factors inducing *IL26* production in resident CD68^+^ macrophage-like synoviocytes are not yet clear and need to be investigated. Studies with human alveolar macrophages have revealed bacterial products such as endotoxins and also environmental toxins (e.g., water-soluble tobacco smoke components) as inducers of *IL26* (22, 38). Potential inducers of *IL26* in CD68^+^ macrophage-like synoviocytes could be damage-associated molecular pattern (DAMP) molecules, such as HMGB1—either released after cell death or actively secreted—activating TLR4 and subsequently NF-κB (39). The latter was shown to be responsible for IL-1β-induced *IL26* production in human memory CD4^+^ T cells (32). Due to the lack of synovial fluid samples from patients with PsA and axSpA, we were not able to further investigate the sources of *IL26* in this compartment, which might differ from the actual synovial tissue. CD3^+^ T cells and fibroblast-like synoviocytes identified by cadherin-11 and CD90 are not involved in the increased expression of *IL26* in the synovium of axSpA and PsA in our study, confirming the findings from SpA synovial fluid by Heftdal et al. (16). This contrasts with the findings from RA joint tissue, where *IL26* co-localizes with CD3, RORγt, and synoviolin (15). However, *IL26* supports Th17 immunity by upregulating IL-17A secretion from Th17 cells and inducing plasticity of memory non-Th17 cells toward Th17 cells through upregulation of IL-1β secretion from macrophages (15). In context of the DNA-binding capacity of *IL26*, it remains to be elucidated if *IL26* in the peripheral joints of SpA patients also represents extracellular traps and, therefore, a further mechanism by which *IL26* perpetuates chronic inflammation. *IL26* has been shown to enhance NET-induced secretion of IL-6 from human monocytes and of CXCL8 from human neutrophils (13). As only a small fraction of *IL26*^+^ synovial cells are CD68^+^ in axSpA and PsA, further detailed investigations of local producers of *IL26* in axSpA and PsA are warranted. Candidates include mainly CD3-negative cell types like ILC3, fibroblast-like synoviocytes and neutrophils. One caveat of our study is the fact that most of the joint samples analyzed were acquired during joint replacement surgery and, therefore, most likely represent a chronic state of inflammation with low activity in axSpA and PsA. In addition, these peripheral samples might not represent the mechanisms of inflammation and bone remodeling in the axial joints of SpA patients. This, however, is a common problem of SpA research, and the previously examined facet joints from SpA patients were also from established, chronic diseases. Nevertheless, *IL26* is also present in the bone marrow of these joints (16).

Despite significantly elevated expression of *IL26* in peripheral joint tissue in axSpA and PsA, we did not observe a relevant increase of *IL26* in the serum of axSpA patients. This is congruent with the results of a previous study on plasma levels of *IL26* in SpA patients (16). However, *IL26* serum levels were significantly increased in PsA and RA patients compared with HCs. This could be explained by the fact that PsA and RA represent inflammatory arthritis with peripheral arthritis, whereas axSpA is predominately an axial disease. In our cohort of axSpA patients, only 27.6% had peripheral disease compared with 95% of PsA patients. The significantly elevated percentages of IL-1R1^+^ Th17 cells in PsA patients identified by chemokine receptor profile might also explain the significantly higher levels of serum *IL26* in this group compared with HCs. Considering the significant enhancement of *IL26* in the peripheral joint tissue of axSpA patients, this points to a distinct role of *IL26* at the site of inflammation in axSpA compared with PsA and RA. Additional local factors involved in this distinct role of *IL26* in axSpA joints need to be identified in the future. Differences between the serum levels of cytokines and local expression are not uncommon and have been described for example for IL-20 in patients with rheumatoid arthritis (40). This discrepancy has also been observed in patients with IBD, where increased protein and mRNA expression levels of *IL26* in the gut were evident compared with HCs, but *IL26* serum levels and *IL26* expression in PBMCs were lower (41). This would fit with our observation of local *IL26* attributable to tissue-resident CD68^+^ macrophage-like synoviocytes in SpA and not extravasated CD3^+^ T cells. A recent study in HCs demonstrated a large proportion of **IL26*^+^IL-17A^−^* cells within circulating Th17 cells (identified through chemokine receptor staining) via PrimeFlow (42). This population seems to be a Th17 intermediate as it differentiates into *IL-17A^+^* Th17 cells upon TGF-β1 exposure. Such intermediates and the described differentiation were also shown in psoriatic skin lesions. Therefore, the lack of *IL26*^+^CD3^+^ cells in our peripheral tissue samples could be attributed to a loss of *IL26* expression in chronic or long-standing disease. Additionally, the only slightly elevated percentages of circulating *IL26*^+^ Th17 cells in axSpA and PsA patients approximately corresponded with the results from the serum measurements of *IL26*, further strengthening the local role of *IL26* in SpA. However, the reduced sample number in the PrimeFlow assay of *IL26* (approx. 15 vs. 30 samples) might be responsible for not showing significantly elevated percentages of **IL26*^+^* Th17 cells. Moreover, the slightly increased percentages of *IL26*^+^CD8^+^ T cells in axSpA and PsA could be an alternative source of peripheral *IL26*. This could reflect the fact that, like IL-17A, *IL26* can be produced by different cell types as part of a pathologic type 3 immunity. Nevertheless, we found memory CD4^+^ T cells from axSpA patients to be more prone to *IL26* expression upon encountering typical Th17-favoring cytokine combinations like IL-2, IL-1β, and IL-23 *in vitro*. In addition, circulating CD4^+^ T cells from axSpA and PsA more often expressed **IL26* ex vivo* compared with HCs, being in congruence with previously reported data showing *IL26* expression exclusively in peripheral CD4^+^ T cells (12).

*IL26* could play a major part in the chronicity of inflammation in axSpA and PsA as *IL26* can increase the secretion of IL-1β, IL-6, TNFα, and CCL20 (Th17 cell recruitment) from CD14^+^ myeloid cells as shown from RA synovial fluid (15). In this context, therapies targeting *IL26* could be of great relevance in patients failing current therapies. Due to the employment of JAK1 and TYK2 downstream of the *IL26* receptor, current JAK inhibitors like upadacitinib should reduce *IL26* responses to some extent. However, specifically targeting *IL26* could still have superior effects to current JAK1 or future TYK2 inhibition as several other signaling options have been described for *IL26*. For example, *IL26*/DNA complexes can enter plasmacytoid dendritic cells and activate TLR9 or activate monocytes by inflammasome-mediated activation of the STING (stimulator of interferon genes) pathway (12, 13). It also remains possible that *IL26* alone due to its chemical properties could penetrate the cell membrane and illicit proinflammatory responses (13, 43). This aspect also remains interesting as it is not clear through which pathway *IL26* induces bone mineralization in human osteoblasts (16). So far, the specific receptor chain for *IL26*, IL-20R1, has been detected in human amniotic fluid-derived stem cells, which can be differentiated into osteocytes, and in the mouse osteoblast precursor cell line MC3T3-E1 (44, 45), but not in primary human osteoblasts or osteoblast precursors. So, the exact role and mechanisms of *IL26* in SpA-associated osteoproliferation remain unclear and warrant further investigation. Inhibiting RORγt, one of the key transcription factors of Th17 cells, significantly downregulates the expression of *IL26* in axSpA Th17 cells (46), but unfortunately, none of the candidate compounds has been successfully trialed so far (47). As IL-17A increases *IL26* secretion from RA synovial fibroblasts, IL-17A blockers like sekukinumab could also exert some effects on synovial pathophysiology in axSpA and PsA (15). Monoclonal antibodies targeting *IL26* significantly suppressed imiquimod-induced skin inflammation in human *IL26* transgenic mice without apparent effects on antimicrobial activity (48). In this study, the concentrations of anti-*IL26* mAb used were much lower than the concentrations reported necessary for the antimicrobial effects of *IL26*. This allows for further evaluation of anti-*IL26* mAb as a therapeutic target in inflammatory diseases like psoriasis, PsA and axSpA. As *IL26* secretion from human memory CD4^+^ T cells induced by IL-1β/IL-1R1 was nearly abrogated by pharmacological inhibition of NF-κB activation, this provides a further target for blocking *IL26*-induced pathology assuming a role of the aforementioned **IL26*^+^IL-17^−^* Th17 intermediates in the active or early stages of the disease (32).

In summary, we have shown that different cells are responsible for local and systemic production of *IL26* in axSpA and PsA providing evidence for the first time that CD68^+^ macrophage-like synoviocytes could be a local source of *IL26* in SpA peripheral joints. Regarding its role in chronicity of inflammation and osteoproliferation, *IL26* provides an interesting therapeutic approach for axSpA and PsA by simultaneously targeting the two key modules of its pathophysiology.

### Limitations of the study

Nevertheless, the results of this study must be interpreted bearing a few limitations in mind. The most obvious limitation is the fact that we were unable to obtain corresponding peripheral blood and synovial fluid for the analysis of *IL26* levels and producers. This might have helped to prove that *IL26* is indeed enhanced at the site of joint inflammation in axSpA. However, it has already been shown that the levels of *IL26* are significantly increased in SpA synovial fluid compared with matching plasma (16). Our study unfortunately can also not provide any information on *IL26* expression and producers in axial joints. This is an issue that is hard to overcome as biopsies of axial joints or iliosacral joints are not routinely performed and usually involve CT localization exposing patients to radiation. Another limitation is the small sample size used for phenotyping of *IL26*-expressing T cells and IL-1R1^+^ T cells by flow cytometry. Therefore, a significant enrichment of **IL26*^+^* CD4^+^ or *IL26*^+^CD8^+^ T cells as well as IL-1R1^+^ T cells in axSpA and PsA might not have become evident, despite the clear increase in local *IL26* levels in synovial tissue. Additional studies involving larger numbers of patients are necessary to clarify the role of circulating *IL26*-producing cells. Further limitations are the low disease activity of the PBMC cohort and the long-standing disease phenotype of the synovial tissue samples. We were unable to provide single-cell RNA sequencing of unfixed synovial tissue from patients with active peripheral disease or from affected axial joints. Such data will however be of great interest for further delineating the producers of *IL26* in SpA in the future.

## Data availability statement

The raw data supporting the conclusions of this article will be made available by the authors, without undue reservation.

## Ethics statement

The studies involving humans were approved by 100/18 S, Ethical Committee of the Faculty of Medicine, Technical University Munich and 06/Q1606/139 Oxford University Hospitals NHS Foundation Trust. The study has been registered within the German Clinical Trials Register (DRKS00014672). The studies were conducted in accordance with the local legislation and institutional requirements. The participants provided their written informed consent to participate in this study.

## Author contributions

AH: Conceptualization, Formal analysis, Funding acquisition, Investigation, Methodology, Supervision, Validation, Visualization, Writing – original draft, Writing – review & editing. AO: Formal analysis, Investigation, Visualization, Writing – original draft. QB: Writing – review & editing, Resources. HM-F: Writing – review & editing, Resources. GL: Writing – review & editing, Resources. MW: Writing – review & editing, Resources. FW: Writing – review & editing, Resources. HM: Writing – review & editing, Resources. DS: Writing – review & editing, Resources. PB: Supervision, Writing – review & editing, Resources. UH: Resources, Writing – review & editing. MA: Writing – review & editing, Resources. PM: Resources, Supervision, Writing – review & editing. CS: Resources, Supervision, Writing – review & editing.
