# Supplementary material for: Increased interleukin-26 in the peripheral joints of patients with axial spondyloarthritis and psoriatic arthritis, co-localizing with CD68-positive synoviocytes
Source: Front Immunol. 2024 May 10;15:1355824. doi: 10.3389/fimmu.2024.1355824 (PMC11127564; doi:10.3389/fimmu.2024.1355824)
Supplement: Supplementary file 1 [file DataSheet_1.pdf]

Supplementary Material

Supplementary Material and Methods

Patient samples

Venous blood (max. 50 ml) and synovial tissue were obtained from patients with axSpA (n=38), PsA (n=23), RA (n=22) and HC (n=40), following informed written consent, with ethical permission (100/18 S, ethical committee of the faculty of medicine, Technical University Munich and 06/Q1606/139 Oxford University Hospitals NHS Foundation Trust). The study has been registered within the German Clinical Trials Register (DRKS00014672). Healthy donors were sex and age matched to the axSpA group. AxSpA patients met the modified New York criteria, PsA patients the CASPAR (classification criteria for psoriatic arthritis) criteria and RA patients the American college of rheumatology/European league against rheumatism 2010 criteria. All research was performed in accordance with the relevant guidelines and regulations including the Helsinki Declaration.

Serum was isolated from a 4.7 ml Serum tube (Sarstedt), aliquoted and stored at -80°C till analysis.

Cell Purification and Cell Culture

Peripheral blood mononuclear cells (PBMC) were isolated by Ficoll density-gradient centrifugation from prefilled tubes (4x8 mL, CPT tube, BD). CD4+ T cells were then negatively and CD14+ positively selected via magnetic beads (Biolegend, > 90.96% purity on average) and cultured in Roswell Park Memorial Institute medium (RPMI-1640; Sigma-Aldrich) supplemented with 1% penicillin-streptomycin (P/S, 10.000 units, 10 mg/ml; Sigma-Aldrich) and 10 % fetal bovine serum (FCS, Sigma-Aldrich) at 37°C and 5 % CO_2 in a humidified atmosphere. Cells were cryopreserved in FCS with 10% DMSO (Sigma-Aldrich) in liquid nitrogen till analysis.

CD4+ T cells were cultured in 750 µl RPMI 10% FCS and 1% P/S with anti-CD2/CD3/CD28 beads (Miltenyi Biotec) at a ratio of 1 bead per 10 cells in 48-well plates ($1.33x10^6$ cells per well) to induce IL-26 production for 6 days: Recombinant human IL-2 (10 IU/ml; Peprotech), IL-1β, IL-6 and IL-23 (all at 20 ng/ml; Peprotech), TNFα (10 ng/ml, Peprotech). 50% of cytokine-supplemented medium was exchanged after 3 days. The following cytokine combinations were used: IL-2, IL-2 + IL-1β + IL-23, IL-2 + IL-1β + IL-23 + TNFα and in a few samples IL-2 + IL-1β + IL-23 + IL-6 (with beads 1 in 20 cells and IL-2 at 100 IU/ml).

CD14+ monocytes were cultured at a density of $1x10^6$ cells per ml on 6-well plates in RPMI 10% FCS for 3 and 6 days in the presence of LPS at 10 µg/ml (InvivoGen).

Synovial tissue acquisition and preparation

Synovial tissue was obtained during orthopaedic surgery or arthroscopy and directly fixed in 4% Formaldehyde for 48h at room temperature. After fixation, tissue was processed using a Leica ASP300S tissue processor and embedded in paraffin wax. Tissue was embedded in paraffin and cut into 1.5 µm sections using a rotary RM2145 microtome (Leica) onto adhesive glass slides.

ELISA

Serum levels of IL-26 were analysed with a modified IL-26 ELISA kit (Elabscience) with the intention to minimize false results through interfering antibodies (1). Therefore, samples were preincubated with heat-aggregated bovine IgG (10µg/ml, Jackson ImmunoResearch Laboratories) and biotinyl-tyramide (1 mM; Tocris Bioscience) was used for signal amplification.

Immunohistochemistry

For antigen retrieval, slides were baked at 60°C for 60 minutes and tissue sections were taken through deparaffinisation and target retrieval steps (heat mediated antigen

retrieval at pH 9 using a TRIS-EDTA buffer at 97±1°C for 20 min) manually. Antibody staining was performed using the DAKO Envision+ System HRP Kit (Dako) according to the manufacturer´s protocol. Endogenous Peroxidase activity was blocked with Peroxidase Block and unspecific binding with Serum-Free Protein Block (both from DAKO). The primary antibody (anti-IL-26, clone MAB1375, mouse IgG2B, R&D) and isotype control (clone MAB0042, R&D) were diluted in 5% normal goat serum (Jackson ImmunoResearch Laboratories) to 10 µg/ml final concentration and incubated over night at 4°C. After incubation with HRP-conjugated, polymerised goat-anti-mouse secondary antibodies for 30 min at RT antibody binding was visualized using liquid 3,3'-diaminobenzidine (DAB)+ substrate working solution (DAKO) and haematoxylin counterstain (Carl Roth). After staining, slides were taken through graded industrial methylated spirit and xylene, and mounted in Roti-Histokitt II mounting medium (Carl Roth).

Image acquisition and quantitative analysis for immunohistochemistry

Images were acquired on a Whole-Slide Scanner NanoZoomer 2.0-RS C10730 (Hamamatsu) using the NDP.Scan software in a 40x optical resolution. Digitalised slides were then divided into single images of 300 DPI at a 20x magnification. Automated semiquantitative analysis was performed with an open-source plug in ImageJ (2). This tool separates the DAB from the Hematoxylin channel and then performs a pixel analysis of the DAB channel for each single image. The pixels are categorized as percentages of high positive, positive, low positive and negative relative to the total pixels of all combined single images.

Immunofluorescence

The protocol is adapted from Dakin S et al. with incorporation of the Tyramide SuperBoost Kit (Invitrogen) (3). After antigen retrieval steps, tissues were blocked with peroxidase block (DAKO) and subsequently with blocking buffer for 1h in a humid

chamber at RT (2.5% horse serum for blocking for Cadherin-11 and CD90). Sections were incubated with the IL-26 antibody (MAB1375, R&D) diluted to 10 µg/ml in 10% normal goat serum in TBST over night at 4°C. Sections were washed 3 times with TBST and incubated with one drop of goat anti-mouse Poly-HRP antibody for 1 hour at RT. After washing, sections were incubated with AF594 tyramide reagent for 10 min at RT in the dark. Then the reaction was terminated and the slides washed in TBST. For double labelling with additional primary mouse antibodies (CD163 - EDHU-1, Bio-Rad, and SMA - 1A4, DAKO-Agilent) or polycloncal goat and sheep antibodies (Cadherin-11 and CD90, R&D Systems) slides were then again subjected to antigen retrieval steps. After blocking with peroxidase block and serum-free protein block (DAKO) or 2.5% horse serum (for the polyclonal antibodies) for 10 and 30 min at RT, slides were incubated with the respective concentrations of primary antibodies in 10% normal goat serum (or animal-free blocking buffer) in TBST (see Supplementary Table 1) over night at 4° in a humid chamber. Slides were washed again and incubated with secondary antibodies 1:100 in 10% normal goat serum in TBST (goat anti-mouse-FITC IgG, polyclonal and goat anti-rabbit-FITC IgG, polyclonal, Dianova) for 1h at RT. For Cadherin-11 and CD90 staining slides were incubated with horse anti-goat Poly-HRP antibody (Vector Laboratories) or donkey anti-sheep Poly-HRP antibody (R&D Systems) for 30 min at RT. Nuclear counterstaining was achieved using DAPI-containing mounting medium (VectaShield, Vector Laboratories) and slides were sealed and stored at 4°C until image acquisition. For negative controls the primary antibody was substituted with matched isotype controls (see Supplementary Table S1). Immunofluorescence images were acquired on a Leica SP5 confocal microscope using a 20x oil immersion objective (NA=0.7). The fluorophores DAPI, Alexa Fluor 488 and Alexa Fluor 594 were excited using the 405nm, 488nm and 595nm laser lines respectively. To minimize bleed-through, AF488 and AF594 channels were acquired sequentially.

Averaging was set to 1 and the pinhole was set to approximately 1 Airy unit. Images were analysed using LAS AF Lite Software (Leica). Images of Cadherin-11 and CD90 co-staining with IL-26 were acquired on a BZ-9000 inverted fluorescence microscope (Keyance) with an 20x and 40x objective. Images were analysed using BZ-II Viewer (Keyance).

Flow Cytometry

For surface phenotyping of IL-1R1 and chemokine receptors PBMC were incubated in RPMI 10% FCS with IL-2 (1.000 IU/ml; Peprotech) at a density of $1x10^6$ cells per ml on a U-bottom 96-well plate over night at 37°C and 5% CO_2. Staining antibodies and dilutions are shown in Supplementary Table S2. The CXCR3 staining was performed prior to all other staining for 20 min at 37°C and 5% CO_2 in the dark. All other antibodies were stained together for 30 min at RT. Cells were washed (DPBS 1% FCS; Sigma) and fixed for 30 min on ice (FACS wash with 2% Formaldehyde; Sigma) prior to storage at 4°C in the dark. Samples were acquired on a Cytoflex S (Beckman Coulter) within 1-2 h and analysis was done with FlowJo software version 10.8.1 (Treestar).

PrimeFlow Assay

The PrimeFlow RNA Assay kit (Invitrogen) was used for detection of *IL26*-positive cells via Flow Cytometry in combination with conventional surface and intracellular cytokine staining according to manufacturer´s protocol. PBMC were plated on a U-bottom 96-well plate at a density of $5x10^6$ cells/well in RPMI 10% FCS. Phorbol myristate acetate (100 ng/ml; Sigma-Aldrich) and ionomycin (1 µg/ml; Sigma-Aldrich), in the presence of Golgiplug (Brefeldin A) and Golgistop (Monensin) (both from BD Biosciences diluted according to manufacturer’s instructions), were used for stimulation for four hours at 37 °C in 5% CO_2. All further steps were performed on a V-bottom 96-well plate. The respective antibodies for surface and intracellular cytokine staining and mRNA probes

are detailed in Supplementary Table S3. The anti-CD4 antibody was also added to the intracellular cytokine antibody cocktail, as staining was affected by the fixation step. Fluorescence minus one (FMO) controls (IL-17A and *IL26*) and positive controls (*RPL13A*) were included in each experiment. Flow cytometry was performed within 24h on a Cytoflex S or LX (Beckman Coulter), which was calibrated before every experiment with CytoFLEX Daily QC Fluoropheres (Beckman Coulter). The data were analysed using FlowJo software version 10.8.1 (Treestar).

qPCR

CD4+ T cells (1×10^6) were cultured on 48-well plates as described above for 6 days. CD4+ T cells were subjected to total RNA isolation with the Nucleospin RNA Mini Kit (Macherey-Nagel) on day 0 or day 6. For quantitative PCR (qPCR), RNA was reverse transcribed with the High Capacity cDNA Reverse Transcription Kit (Applied Biosystems). TaqMan probes for transcripts encoding *IL26*, *IL17A*, *IL22*, *IFNG*, *IL4*, *RORC* and *IL1R1* (see Supplementary Table S4 for detailed information) were used with Luna Universal qPCR Master Mix (New England BioLabs) on a Lightcycler 480 II Real-Time PCR System (Roche). Assays were performed in triplicate, and gene expression levels were normalized to ACTB (β-actin) and RPL13A with ΔΔCt calculations according to Taylor et al. (4).

Statistical analysis

GraphPad Prism software version 5 was used for statistical analysis. When comparing multiple groups with normal distribution of independent, continuous variables a One-way analysis of variance (ANOVA) with a Dunnett post-test for correction was used. When comparing multiple groups with normal distribution of dependent, continuous variables a Repeated Measures ANOVA with a Dunnett post-test for correction was used. When comparing multiple groups with non-normal distribution of independent,

continuous variables a Kruskal-Wallis test with a Dunnett post-test for correction was used. When comparing two groups with non-normal distribution of dependent, continuous variables a Wilcoxon matched-pairs signed rank test was used. Correlation analysis of normally distributed, continuous variables was performed according to Pearson. Significance was defined as $p \leq 0.05$.

Supplementary References

1. Kragstrup TW, Vorup-Jensen T, Deleuran B, Hvid M. A simple set of validation steps identifies and removes false results in a sandwich enzyme-linked immunosorbent assay caused by anti-animal IgG antibodies in plasma from arthritis patients. Springerplus. Dezember 2013;2(1):263.

2. Varghese F, Bukhari AB, Malhotra R, De A. IHC Profiler: an open source plugin for the quantitative evaluation and automated scoring of immunohistochemistry images of human tissue samples. PLoS One. 2014;9(5):e96801.

3. Dakin SG, Martinez FO, Yapp C, Wells G, Oppermann U, Dean BJF, u. a. Inflammation activation and resolution in human tendon disease. Sci Transl Med [Internet]. 28. Oktober 2015 [zitiert 10. Januar 2023];7(311). Verfügbar unter: https://www.science.org/doi/10.1126/scitranslmed.aac4269

4. Taylor SC, Nadeau K, Abbasi M, Lachance C, Nguyen M, Fenrich J. The Ultimate qPCR Experiment: Producing Publication Quality, Reproducible Data the First Time. Trends Biotechnol. Juli 2019;37(7):761–74.

Supplementary Figure Legends

Supplementary Figure S1: Weak positive correlation between Serum IL-26 concentration and CRP in axSpA

Correlation analysis was performed between CRP and serum IL-26 levels measured by ELISA in 25 axSpA, 16 PsA and 14 RA patients. r: pearson's correlation coefficient, r^2: coefficient of determination, $p<0.05$ considered significant.

Supplementary Figure S2: Gating strategy used for Prime Flow and surface marker characterisation of Th17 cells

A) Gating strategy for identification of live *IL26*+CD4+ and *IL26*+CD161+CD26+CD4+ cells from PBMC from axSpA, PsA patients and HC in the Prime Flow assay. B) Gating strategy for identification of live IL-1R1+ Th17 cells (CCR4+CXCR3-CCR6+CD161+CD4+CD45RO+) and IL-1R1+CD161+CD26+CD4+CD45RO+ cells from PBMC from axSpA, PsA patients and HC with surface marker staining.
Plots in A) and B) are from representative samples.

Supplementary Figure S3: Increased expression of *IL26* in CD4+ T cells from axSpA and PsA patients parallels expression of other Th17-type markers

A) *Ex vivo* expression of *IL26* in purified CD4+ T cells from patients (axSpA n=19, PsA n=18, RA n=13) and HC (n=20) was assessed by qPCR. B) Total CD4+ T cells from HC (n=20), axSpA (n=19), PsA (n=18) and RA n=13) were incubated with IL-2, IL-1β and IL-23 *in vitro* for 6 days and expression of *IL4, IL1R1, IL22* and *IFNG* was analysed by qPCR in triplicates. Expression was normalized to two control genes (*ACT* and *RPL13A*) and is shown as fold expression in relation to HC. C) CD4+ T cells from the same cohort as in B) were stimulated with different Th17-favouring cytokine combinations for 6 days *in vitro* and fold expression (triplicates each) of the respective genes

is shown in relation to basal stimulation with IL-2 after normalizations for *ACT* and *RLP13A*.

Statistical analysis: mean ± SEM, *p<0.05, **p<0.01, ***p<0.001 (B: one-Way ANOVA and C: repeated measures ANOVA followed by Dunnett post-test for multiple comparisons).

Supplementary Figure S4: Co-localization of IL-26 and CD68+ macrophage-like synoviocytes in peripheral PsA synovial tissue

Representative immunofluorescence image of peripheral joint tissue from PsA stained for IL-26 (red) and SMA/CD68/CD163 (green). Blue shows DAPI nuclear stain. Scale bar 50 µm. Arrows indicate cells of interest for co-localization of respective cell surface markers and IL-26.

Supplementary Tables

Supplementary Table S1 Antibodies for Immunofluorescence

Antigen (human)	Clone	Host	Antibody type	Supplier	Concentration
IL-26	MAB1375	Mouse	IgG2B, monoclonal	R&D	10 µg/ml
IL-26 Isotype control	MAB0042	Mouse	IgG2B, monoclonal	R&D	10 µg/ml
CD3	EP4426	Rabbit	IgG, monoclonal	Abcam	2,32 µg/ml
CD3 Isotype control	AB-105-C	Rabbit	IgG, monoclonal	R&D	2,32 µg/ml
CD163	EDHU-1	Mouse	IgG1, monoclonal	Biorad	1,25 µg/ml
CD163 Isotype control	G3A1	Mouse	IgG1, monoclonal	CST	1,25 µg/ml
CD68	D4B9C	Rabbit	IgG, monoclonal	CST	365 ng/ml
CD68 Isotype control	AB-105-C	Rabbit	IgG, monoclonal	R&D	365 ng/ml
SMA	1A4	Mouse	IgG2a, monoclonal	Agilent	700 ng/ml
SMA Isotype control	eBM2a	Mouse	IgG2a, monoclonal	TFS	700 ng/ml
Cadherin-11		Goat	polyclonal	R&D	2 µg/ml
Nomal goat IgG		Goat	polyclonal	R&D	2 µg/ml
CD90		Sheep	polyclonal	R&D	1 µg/ml
Normal sheep IgG		Sheep	polyclonal	R&D	1 µg/ml
Anti-mouse FITC (sec. Antibody)	-	Goat	IgG, polyclonal	Dianova	1:100 (Dilution)

Anti-rabbit FITC (sec. Antibody)	-	Goat	IgG, polyclonal	Dianova	1:100 (Dilution)

Supplementary Table S2 Antibodies for Flow Cytometry IL-1R1 surface stain

Antibody target	Fluoro-chrome	Clone	Supplier	Dilu-tion	Incubation condition
Fixable Vi-ability Dye	eFluor 780	na	eBioscience	1:400	30 minutes at RT in the dark
CD3	BV510	SK7	BioLegend	1:50	30 minutes at RT in the dark
CD4	BV605	OKT4	BioLegend	1:25	30 minutes at RT in the dark
CD8	ECD	SFCI21Thy2D3	Beckman Coulter	1:50	30 minutes at RT in the dark
CD26	PeCy7	BA5b	BioLegend	1:50	30 minutes at RT in the dark
CD161	BV421	HP-3G10	BioLegend	1:25	30 minutes at RT in the dark
IL-1R1	PE	FAB269F	R&D Sys-tems	1:10	30 minutes at RT in the dark
CCR4 (CD194)	APC	L291H4	BioLegend	1:25	30 minutes at RT in the dark
CCR6 (CD196)	AF488	G034E3	BioLegend	1:100	30 minutes at RT in the dark
CD45RO	PerCPCy5.5	UCHL1	BioLegend	1:100	30 minutes at RT in the dark
CXCR3 (CD183)	BV785	G025H7	BioLegend	1:50	20 minutes at 37°C, 5% CO_2

Supplementary Table S3 Antibodies for Prime Flow Assay

Antibody target	Fluorochrome	Clone	Supplier	Dilution	Incubation condition
Fixable Viability Dye	eFluor 780	na	eBioscience	1:400	30 minutes on ice in the dark
CD3	BV510	SK7	BioLegend	1:50	30 minutes on ice in the dark
CD4	BV605	OKT4	BioLegend	1:25	30 minutes on ice in the dark
CD8	ECD	SFCI21Thy2D3	Beckman Coulter	1:50	30 minutes on ice in the dark
CD26	PeCy7	BA5b	BioLegend	1:50	30 minutes on ice in the dark
CD161	BV421	HP-3G10	BioLegend	1:25	30 minutes on ice in the dark
IL-17A	FITC	eBio64DEC1	eBioscience	1:50	IC-Stain: 30 minutes on ice in the dark
IFNγ	AF700	B27	BD	1:50	IC-Stain: 30 minutes on ice in the dark
IL26/RPL13A	AF647	mRNA target probes	Invitrogen	1:40	IC-Stain: 2h 40±1°C, 5% CO_2 in the dark

Supplementary Table S4 probes for qPCR

Gene	Probe (FAM)	Assay number	Supplier	Concentration Probe/Primer
IL17A	6-FAM/ZEN/IBFQ	Hs.PT.58.2545178	Integrated DNA Technologies, Inc.	5µM/10µM
ACTB	6-FAM/ZEN/IBFQ	Hs.PT.39a.22214847	Integrated DNA Technologies, Inc.	5µM/10µM
IFNG	6-FAM/ZEN/IBFQ	Hs.PT.58.3781960	Integrated DNA Technologies, Inc.	5µM/10µM
RPL13A	6-FAM/ZEN/IBFQ	Hs.PT.58.47294843	Integrated DNA Technologies, Inc.	5µM/10µM
IL26	6-FAM/ZEN/IBFQ	Hs.PT.58.4002576	Integrated DNA Technologies, Inc.	5µM/10µM
IL1R1	6-FAM/ZEN/IBFQ	Hs.PT.58.40825009.g	Integrated DNA Technologies, Inc.	5µM/10µM
IL4	6-FAM/OQ-A	HSAP_NM_000589_1	Sigma-Aldrich	5µM/10µM
RORC	6-FAM/OQ-A	HSAP_NM_001001523_1	Sigma-Aldrich	5µM/10µM
IL22	6-FAM/OQ-A	HSAP_NM_020525_1	Sigma-Aldrich	5µM/10µM

Supplementary Table S5 log2 normalized expression for Fig. 3A and Fig. S3C

Gene	HC				axSpA				PsA				RA			
	1)*	2)*	3)*	4)*	1)*	2)*	3)*	4)*	1)*	2)*	3)*	4)*	1)*	2)*	3)*	4)*
IL26	-0,14	0,37	0,55	-0,61	1,41	1,99	2,82	-0,24	1,70	2,80	3,13	0,70	-0,19	1,93	1,91	-2,32
	-0,22	3,27	3,18	-0,01	-0,61	-0,29	0,78	-0,56	1,07	1,88	2,00	-0,33	-4,03	-3,18	-2,81	-0,60
	1,07	3,06	3,03	-1,26	0,71	2,15	2,41	1,86	0,66	0,80	0,86	0,31	-1,38	-0,57	0,44	0,27
	0,56	2,44	2,50	0,97	-0,83	0,90	0,91		-1,04	-0,11	-0,11	-1,47	-0,96	0,01	0,30	2,05
	3,14	4,09	3,65		-0,32	1,62	1,72		-0,88	2,17	0,77	2,51	-2,31	-1,45	-1,17	3,13
	1,01	3,36	2,42		0,11	1,32	1,26		2,03	2,47	2,08		-0,08	0,87	0,14	2,22
	1,38	3,19	2,84		0,82	1,77	1,32		1,41	1,54	1,73		0,19	5,12	2,49	2,04
	0,66	-1,60	0,90		-0,57	1,27	2,45		-1,13	0,78	0,67		1,55	3,12	2,28	1,68
	1,09	2,45	2,66		1,15	1,64	2,36		0,09	-0,15	0,26		2,06	2,54	2,68	
	0,49	2,35	2,06		1,89	2,72	2,60		0,12	1,42	1,12		3,60	3,17	3,42	
	0,45	1,73	2,15		1,24	2,40	2,37		1,59	2,05	2,00		1,53	2,01	1,82	
	0,02	1,42	1,07		0,01	1,19	1,20		-1,75	-0,11	-0,28					
	-0,73	0,82	1,56		-1,80	0,85	0,89		0,01	0,05	0,78					
	-0,40	0,35	0,95		0,10	-0,43	-0,48		-2,10	-1,85	-1,59					
	-1,53	0,16	0,23		-0,48	-0,27	-0,34		-3,18	-2,96	-2,61					
	-0,98	0,20	-0,34		-4,36	0,85	0,11		1,41	2,71	0,98					
	-2,84	-1,34	-1,31		0,19	0,77	0,94									
	-3,31	-1,94	-1,67		-0,02	0,94	1,18									
	0,29	1,06	1,24		1,35	1,92	1,99									
IL17A	2,84	3,94	3,52	-2,38	3,46	5,22	5,67	0,47	1,75	4,11	4,05	1,90	-2,61	-0,25	-0,22	-2,52
	-2,11	3,41	3,32	2,40	3,62	4,60	4,37	-1,35	0,59	1,79	2,13	0,16	-3,47	-2,99	-3,46	-0,44
	1,12	3,96	4,06	1,91	-1,73	1,79	1,65	4,47	0,02	1,73	2,19	1,01	-0,91	-0,85	0,29	1,62
	0,37	3,07	3,19	0,94	-2,40	-0,05	-0,16		0,30	1,45	1,50	-0,33	-2,11	-0,26	-0,16	3,49
	1,78	6,23	6,28		0,47	3,46	3,56		-3,83	0,27	-1,38	6,06	-1,36	-1,13	-1,29	5,21
	2,31	5,05	4,53		1,92	3,33	3,48		1,85	2,98	2,83		0,08	1,24	0,96	5,16
	1,41	4,28	3,69		-1,01	0,80	0,80		2,14	3,06	3,15		1,53	6,55	3,23	3,53
	1,92	3,60	5,02		-1,18	2,18	3,22		-1,85	1,39	1,47		2,27	4,71	4,75	4,35
	1,59	3,37	4,42		1,79	2,88	2,49		-0,02	-0,37	0,18		2,90	5,15	5,26	
	-0,85	3,33	2,77		1,45	2,59	2,86		-0,26	1,96	1,81		3,68	4,03	3,23	
	-1,19	0,97	0,84		0,06	2,48	2,82		1,94	3,07	3,22					
	-1,03	0,87	0,80		-2,15	-0,05	0,15		-3,04	1,28	-0,27					
	-1,04	1,45	1,68		-0,47	1,67	2,18		-0,62	0,69	-0,14					
	-1,35	1,34	0,85		-1,21	1,26	0,75		-1,05	0,23	0,18					
	-0,93	0,45	-0,39		0,16	1,31	0,95		-1,77	0,42	-0,36					
	-0,15	1,67	2,10		-2,52	1,32	1,19		3,84	5,49	3,57					

	0,01	1,96	2,59		0,46	2,52	2,69									
	-2,72	-0,84	-1,38		-2,67	0,87	0,97									
	-1,98	0,92	0,88		1,93	4,00	3,91									
IL4	2,10	1,57	0,28	-4,23	3,08	2,64	2,33	-2,01	-2,17	-0,85	0,82	1,47	-3,29	-3,12	-3,11	-0,19
	2,52	0,96	0,20	-0,80	1,50	1,92	1,82	-2,04	0,02	0,71	0,48	-1,19	-0,72	-0,99	-1,32	-0,04
	-0,13	-0,19	-0,65	1,99	-0,16	-0,57	-0,81	0,91	-0,34	-0,94	-0,35	0,78	-1,36	-2,80	-1,68	-1,51
	1,97	0,77	0,36	-0,59	-0,44	-0,72	-0,57		-0,03	0,50	-1,70	1,49	-1,85	-1,38	-2,62	-0,22
	-0,33	1,04	-0,89		0,68	0,26	-0,49		-0,12	-0,88	-1,84	7,10	-2,10	-2,97	-2,24	0,65
	0,14	-0,22	-0,58		1,09	0,17	0,11		-0,58	0,54	-0,16		-1,79	-2,05	-2,45	0,85
	3,17	0,68	2,03		-0,73	-1,18	-1,90		1,05	1,07	0,62		1,19	3,85	1,05	3,12
	1,46	-1,31	-0,47		0,65	0,37	1,15		-1,24	-0,05	-0,45		0,83	0,72	0,78	3,20
	0,13	-0,12	-0,74		-0,04	-0,15	0,35		-1,80	-0,38	-0,85		1,33	0,86	1,45	
	-0,74	-1,96	-1,34		0,45	0,60	0,19		1,05	0,93	0,66		3,10	1,40	4,50	
	-0,59	-1,00	-1,88		-1,49	-2,08	-0,58		-0,82	0,17	-1,23		4,67	4,05	3,22	
	-2,26	-2,46	-3,03		-2,53	-2,34	-2,12		-0,58	-0,08	-0,68					
	-3,30	-4,10	-0,80		0,72	-0,41	-0,28		-0,29	-0,43	-0,56					
	-1,09	-1,87	-1,98		-1,18	-3,35	-2,01		1,36	1,52	1,72					
	-0,74	-1,98	-1,59		-3,04	-3,75	-3,28		-1,72	0,53	0,75					
	2,03	2,51	2,13		0,12	0,18	0,03		6,21	7,19	3,94					
	-3,61	0,38	0,60		0,12	-0,23	-0,32									
	-0,74	-1,68	-1,66		0,69	-0,79	-0,52									
					0,51	0,56	-0,52									
IL1R1	3,00	2,26	1,10	-1,28	3,02	2,91	3,60	-1,42	1,50	1,26	1,37	-1,18	-0,12	-1,68	-1,44	-2,00
	1,11	0,89	0,44	-0,83	2,82	2,34	2,04	-1,43	0,41	1,24	0,96	-0,90	-1,83	-2,72	-2,83	-1,29
	0,19	0,93	0,62	0,23	0,83	1,21	0,59	-1,48	-0,40	-1,11	-0,73	-0,73	-3,95	-3,05	-1,76	-1,41
	0,75	1,06	0,81	-0,99	0,34	-0,21	0,29		0,10	-0,18	-0,96	0,75	-2,08	-1,62	-2,40	0,89
	1,98	3,46	3,18		0,96	0,82	0,99		-1,78	-0,56	-2,34	4,93	-2,14	-2,40	-1,80	0,70
	1,66	2,48	2,21		2,28	2,00	1,54		-0,57	-0,69	-0,50		-0,84	-1,37	-1,57	1,42
	0,15	0,60	0,58		0,58	0,97	0,20		-1,40	-1,38	-1,43		1,85	4,37	1,45	2,18
	2,67	0,05	1,49		-1,18	1,64	3,16		-1,39	-1,26	-1,83		0,65	0,54	0,60	1,83
	0,07	0,02	0,66		0,95	0,92	1,13		-0,20	-0,14	-0,34		2,74	2,11	1,89	
	0,01	0,30	0,26		0,98	0,55	0,90		-0,26	1,31	-0,46		2,29	0,72	3,28	
	-1,09	-1,03	-1,25		0,26	-0,15	0,20		-0,74	-1,22	-0,99		3,44	2,54	1,72	
	-0,35	-1,64	-0,91		-1,93	-2,11	-1,83		-1,07	-1,47	-1,13					
	-1,09	-1,31	-1,89		-0,94	-1,60	-1,29		1,03	1,17	0,39					
	-2,16	-1,58	-1,31		-0,90	-1,94	-1,31		0,97	-0,63	0,32					
	-5,45	-1,60	-1,88		-1,00	-1,29	-1,23		3,82	4,27	1,49					
	-0,88	-1,32	-1,39		-1,79	-1,18	-1,62									
	0,25	0,87	0,91		-2,04	-2,37	-2,37									
	-0,81	-1,11	-1,76		-1,72	-2,33	-2,26									
	-0,01	-0,81	-0,97		-1,51	-1,75	-1,94									

RORC	0,02	0,39	-0,10	-0,68	-0,49	-0,24	-0,51	-0,71	2,00	2,20	2,10	-0,32	1,42	0,89	0,64	-1,89
	-1,58	0,59	0,01	-1,19	-0,10	-0,11	0,25	-0,85	0,86	1,13	0,87		-1,43	-1,07	-1,76	-1,76
	0,84	1,23	1,07	-0,65	1,12	0,68	0,78	1,30	0,65	0,18	0,80	-0,90	-1,18	-2,03	-0,98	-1,37
	0,52	0,58	0,43	-1,18	-0,61	-1,00	-0,78		-1,44	-0,21	-0,25	0,62	-0,24	-0,18	-1,01	-0,64
	1,03	0,83	2,22		0,33	0,35	0,76		-0,63	0,14	-0,70	-0,81	-0,07	-0,57	-0,54	0,60
	0,40	0,86	0,89		1,12	0,52	0,40		1,21	1,32	1,15	1,62	-0,15	-0,17	-0,18	0,87
	0,63	1,46	1,03		0,69	0,70	0,37		0,62	0,14	0,10		0,49	2,59	0,04	0,74
	-0,91	-1,16	-1,15		0,19	-0,17	0,99		0,04	-0,05	-0,07		0,54	0,39	0,23	-1,06
	1,33	1,17	1,63		0,06	-0,48	0,50		-5,19	-1,54	-2,00		1,26	1,02	1,11	
	-0,40	-0,19	-0,11		0,80	0,81	1,22		-0,29	0,53	0,15		-0,64	-0,52	-0,93	
	0,48	0,62	0,86		1,31	1,23	1,13		1,10	2,20	0,63					
	-0,08	-0,29	-0,59		0,26	0,11	0,34		0,94	1,93	0,95					
	1,21	-0,06	0,29		-0,61	-0,86	-0,84		1,25	1,32	1,29					
	-0,25	-0,30	-0,79		-0,88	-1,58	-1,56		-0,67	-1,08	-0,92					
	-1,95	-1,52	-1,72		-0,71	-1,41	-1,21		-2,02	0,37	0,54					
	-0,34	-0,65	-1,13		-2,15	-1,73	-2,03		1,58	2,02	0,60					
	-0,37	0,02	-0,29		-1,35	-0,83	-0,98									
	-0,54	-0,91	-0,53		-0,89	-0,62	-0,83									
	-0,03	-0,59	0,09		1,90	1,53	1,73									
IFNG	2,05	2,82	2,58	-2,80	3,61	3,99	5,34	0,10	-0,16	3,01	3,25	1,25	-5,28	-2,89	-2,94	0,06
	1,82	2,67	2,39	1,06	3,49	4,26	4,58	-3,07	1,42	2,87	2,78	1,75	0,21	0,17	0,32	-0,29
	0,86	3,35	3,67	1,19	-1,56	2,00	2,22	4,57	-2,28	-0,71	-0,71	1,11	-1,88	-1,44	-0,30	-0,51
	0,01	2,63	3,22	-0,85	-1,78	1,46	1,94		-2,41	-1,08	-0,44	2,75	-0,43	0,04	-0,22	2,49
	3,21	3,68	3,78		-0,31	2,92	3,07		-0,85	0,78	-0,97	7,18	-0,67	-0,56	-0,39	3,30
	1,35	2,99	2,57		2,24	2,55	2,76		1,05	2,30	2,79		-1,69	-1,01	-1,07	0,26
	0,58	2,31	1,98		-0,09	2,94	2,81		1,29	3,08	3,01		2,88	5,65	3,84	0,20
	3,58	1,60	3,27		1,00	2,09	3,77		-1,41	0,80	1,21		1,67	3,22	2,89	3,60
	1,06	0,67	2,08		1,17	2,56	3,37		-0,33	0,82	0,63		0,58	0,74	1,14	
	-0,26	2,03	1,56		1,88	3,88	4,80		1,08	2,35	2,43		0,68	2,18	2,98	
	0,11	0,91	1,24		-1,15	1,89	2,32		0,41	3,72	3,13		3,94	3,76	3,55	
	-1,53	0,48	0,90		-0,80	1,34	1,71		-0,30	1,19	0,83					
	-2,09	0,07	1,01		-2,49	-0,17	0,26		-1,62	-0,22	0,13					
	-4,47	-1,30	-0,59		-1,95	-0,84	-1,16		-0,23	2,52	1,82					
	-2,91	-0,57	-0,61		-1,05	0,74	0,64		-0,43	1,83	0,32					
	-1,74	0,31	0,15		-4,01	0,64	-0,03		4,76	6,53	4,14					
	-1,34	0,40	0,97		1,10	2,29	2,19									
	0,27	1,17	1,03		0,40	2,74	3,05									
	-0,56	1,03	-2,09		0,30	4,30	4,28									
IL22	0,48	6,29	5,86	-2,06	2,56	4,25	5,24	-13,2	0,37	2,08	2,36	1,64	-4,27	-1,72	-1,47	-1,27
	1,41	4,17	3,91	2,55	3,78	4,45	4,45	-10,2	0,82	1,67	2,08	1,35	-3,99	-2,54	-2,57	0,77
	2,45	4,55	4,59	1,13	-1,65	2,78	1,88	-6,24	-1,77	-0,95	-0,91	2,26	-0,87	1,38	1,29	1,23
	2,05	4,67	6,11	3,92	-0,87	2,33	2,92		-3,33	-1,50	-1,21	0,52	-0,05	1,00	0,83	2,31

3,05	5,57	5,83	0,64	3,79	4,06	-2,46	-0,68	-0,50	4,97	-0,11	0,32	0,69	4,15
-1,57	6,07	5,78	0,82	2,24	2,10	3,17	3,37	3,85		-0,83	0,29	0,52	2,77
-0,64	3,38	2,66	-0,07	1,24	1,30	-1,45	0,93	0,91		2,50	7,15	4,28	3,30
1,12	2,28	2,52	0,65	1,86	2,09	0,35	-0,14	0,84		2,21	4,97	4,66	2,07
0,52	3,03	3,11	1,05	1,23	2,15	2,78	3,34	3,56		2,03	2,57	2,89	
2,45	5,50	4,75	-1,11	0,96	1,63	3,89	4,21	4,08		3,39	3,08	2,10	
-0,91	0,14	0,39	-1,71	-1,68	-1,16	-2,14	-0,21	0,35					
-2,24	-0,79	-0,81	-2,29	0,19	0,78	-0,68	0,15	0,74					
-2,55	1,59	1,45	-2,44	-0,73	-0,44	-0,22	0,40	0,56					
-1,82	1,17	0,85	-2,31	-1,18	-1,04	-2,30	0,19	-0,59					
-4,18	1,91	2,14	-3,62	1,78	-2,49	2,98	4,57	2,10					
-0,57	1,42	1,32	2,19	2,23	2,49								
-0,81	0,45	1,18	2,46	3,69	3,63								
0,56	1,79	1,88	1,92	3,18	2,90								
1,18	3,13	3,12											

*In vitro stimulation conditions (log2 normalized values in relation to condition 1):

1) IL-2
2) IL-2/IL-1β/IL-23
3) IL-2/IL-1β/IL-23/TNF α
4) IL-2/IL-1β/IL-23/IL-6

Supplementary Table S6 log2 normalized expression for Fig. 3B and Fig. S3B

Gene	HC*	axSpA*	PsA*	RA*
IL26	-0,97	1,52	1,55	-0,42
	1,93	-0,76	0,63	-5,53
	1,72	1,68	-0,45	-2,55
	1,10	0,43	-1,36	-2,91
	2,75	1,15	0,93	-2,34
	2,02	0,85	1,22	-3,79
	1,85	1,30	0,30	-1,48
	-2,94	0,80	-0,46	2,77
	1,11	1,17	-1,40	-0,46
	1,01	2,25	0,17	0,77
	0,39	1,93	0,80	0,19
	0,08	0,72	-1,36	0,83
	-0,52	0,38	-1,20	-0,34
	-0,99	-0,90	-3,92	
	-1,18	-0,74	-3,10	
	-1,14	0,38	-4,21	
	-2,68	0,30	1,46	
	-3,28	0,47		
	-0,28	1,45		
IL17A	1,36	3,60	1,71	-2,65
	0,83	2,98	-0,61	-5,39
	1,38	0,17	-0,67	-5,69
	0,49	-1,67	-0,94	-3,26
	3,65	1,84	-2,12	-2,66

	2,47	1,71	0,58	-3,53
	1,70	-0,82	0,66	-1,17
	1,01	0,56	-1,00	4,15
	0,79	1,25	-2,76	1,05
	0,75	0,97	-0,44	2,31
	-1,61	0,85	0,67	2,74
	-1,71	-1,67	-1,12	2,23
	-1,13	0,05	-1,70	1,63
	-1,24	-0,37	-3,05	
	-2,13	-0,31	-2,17	
	-0,91	-0,30	-1,98	
	-0,62	0,90	3,10	
	-3,42	-0,75		
	-1,66	2,38		
RORC	0,28	0,41	1,43	0,14
	0,48	0,53	0,36	-1,81
	1,12	1,33	-0,59	0,77
	0,47	-0,35	-0,98	-2,78
	0,72	0,99	-0,64	-0,92
	0,75	1,17	0,55	-1,31
	1,35	1,35	-0,63	-0,92
	-1,27	0,48	-0,82	1,85
	1,06	0,17	-2,31	1,20
	-0,30	1,45	-0,24	-0,36
	0,51	1,88	1,43	0,27
	-0,40	0,75	1,16	0,66
	-0,17	-0,21	0,54	-1,27
	-0,41	-0,93	-3,99	

	-1,63	-0,77	-1,85	
	-0,76	-1,08	-0,40	
	-0,09	-0,18	1,25	
	-1,02	0,02		
	-0,70	2,18		
IL4	2,07	2,46	-0,95	-0,86
	1,46	1,74	0,61	1,27
	0,30	-0,75	-1,04	0,41
	1,27	-0,91	0,41	-0,54
	1,54	0,08	-0,98	0,88
	0,28	-0,01	0,44	-0,71
	1,17	-1,36	0,97	0,21
	-0,81	0,19	-0,14	6,11
	0,38	-0,33	-0,47	2,48
	-1,46	0,42	0,83	2,98
	-0,50	-2,27	0,07	3,12
	-1,96	-2,52	-0,18	3,66
	-3,60	-0,59	-0,52	6,31
	-1,37	-3,53	-2,70	
	-1,48	-3,93	1,42	
	3,01	0,00	0,43	
	0,88	-0,42	7,10	
	-1,18	-0,97		
		0,38		
IL1R1	2,13	2,57	0,55	-1,31
	0,76	2,01	0,53	-2,35
	0,80	0,88	-1,82	0,25
	0,92	-0,54	-0,89	-2,69

	3,33	0,49	-1,28	-1,26
	2,35	1,67	-1,40	-2,04
	0,46	0,64	-1,50	-1,00
	-0,08	1,31	-2,09	4,74
	-0,11	0,59	-1,97	0,70
	0,17	0,22	-0,85	0,91
	-1,16	-0,48	0,60	2,47
	-1,77	-2,44	-1,93	1,09
	-1,44	-1,93	-2,19	2,90
	-1,71	-2,27	0,33	
	-1,73	-1,62	0,05	
	-1,46	-1,51	-1,34	
	0,74	-2,70	3,56	
	-1,24	-2,66		
	-0,94	-2,08		
IL22	3,32	2,70	0,02	-4,04
	1,20	2,90	-0,40	-4,85
	1,58	1,23	-3,01	-3,73
	1,71	0,79	-3,56	-0,93
	2,61	2,24	-2,75	-1,32
	3,10	0,69	1,31	-1,99
	0,42	-0,30	-1,13	-2,02
	-0,68	0,50	-2,20	4,84
	0,07	0,31	1,28	3,31
	2,53	-0,31	2,14	2,65
	-2,82	-0,59	-2,28	0,26
	-3,75	-3,22	-1,91	2,31
	-1,37	-1,35	-1,81	0,77

	-1,79	-2,27	-1,66	
	-1,05	-2,73	-1,87	
	-1,54	0,23	2,51	
	-2,51	0,69		
	-1,18	2,15		
	0,17	1,63		
IFNG	1,38	1,67	0,59	-3,82
	1,24	1,94	0,45	-0,76
	1,92	-0,32	-3,13	-1,43
	1,20	-0,85	-3,50	-2,37
	2,25	0,61	-1,64	-0,89
	1,56	0,23	-0,12	-1,49
	0,87	0,63	0,66	-1,94
	0,16	-0,23	-1,62	4,72
	-0,76	0,24	-1,60	1,90
	0,59	1,57	-0,07	2,29
	-0,52	-0,42	1,30	-0,19
	-0,96	-0,98	-1,23	1,25
	-1,36	-2,48	-2,64	2,84
	-2,73	-3,16	-3,52	
	-2,00	-1,57	0,10	
	-1,13	-1,67	-0,59	
	-1,04	-0,03	4,11	
	-0,26	0,43		
	-0,41	1,98		

*In vitro stimulation condition (log2 normalized values in relation to HC cohort):

IL-2/IL-1β/IL-23
