# Supplementary figures and images for: Increased interleukin-26 in the peripheral joints of patients with axial spondyloarthritis and psoriatic arthritis, co-localizing with CD68-positive synoviocytes
Source: Front Immunol. 2024 May 10;15:1355824. doi: 10.3389/fimmu.2024.1355824 (PMC11127564; doi:10.3389/fimmu.2024.1355824)

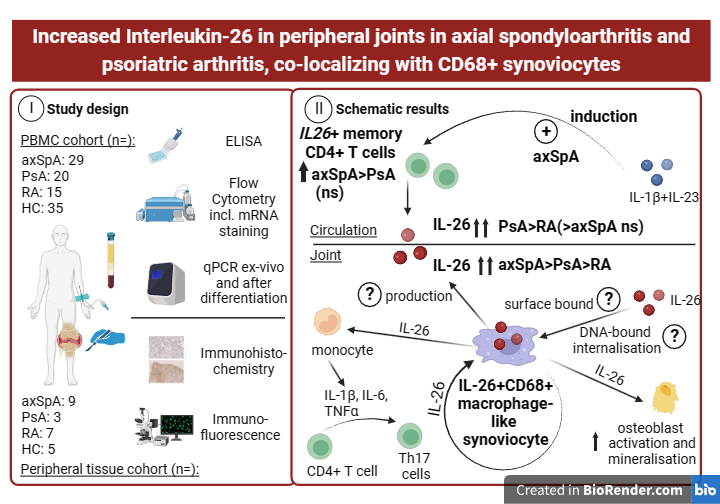

Supplement: Supplementary file 2 [file DataSheet_1.zip › graphical abstract.PNG]

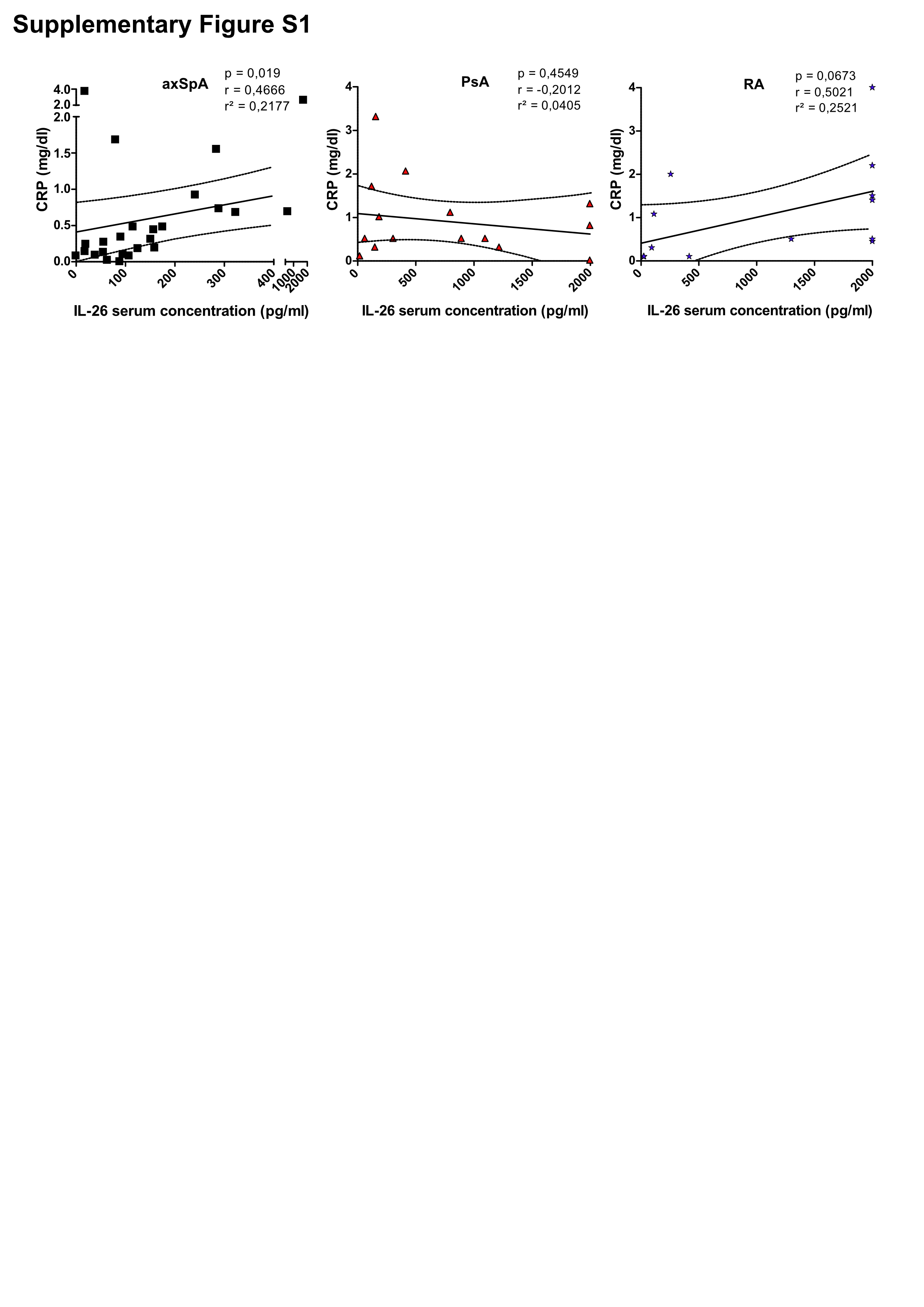

Supplement: Supplementary file 2 [file DataSheet_1.zip › Supplementary Figure 1.TIFF]

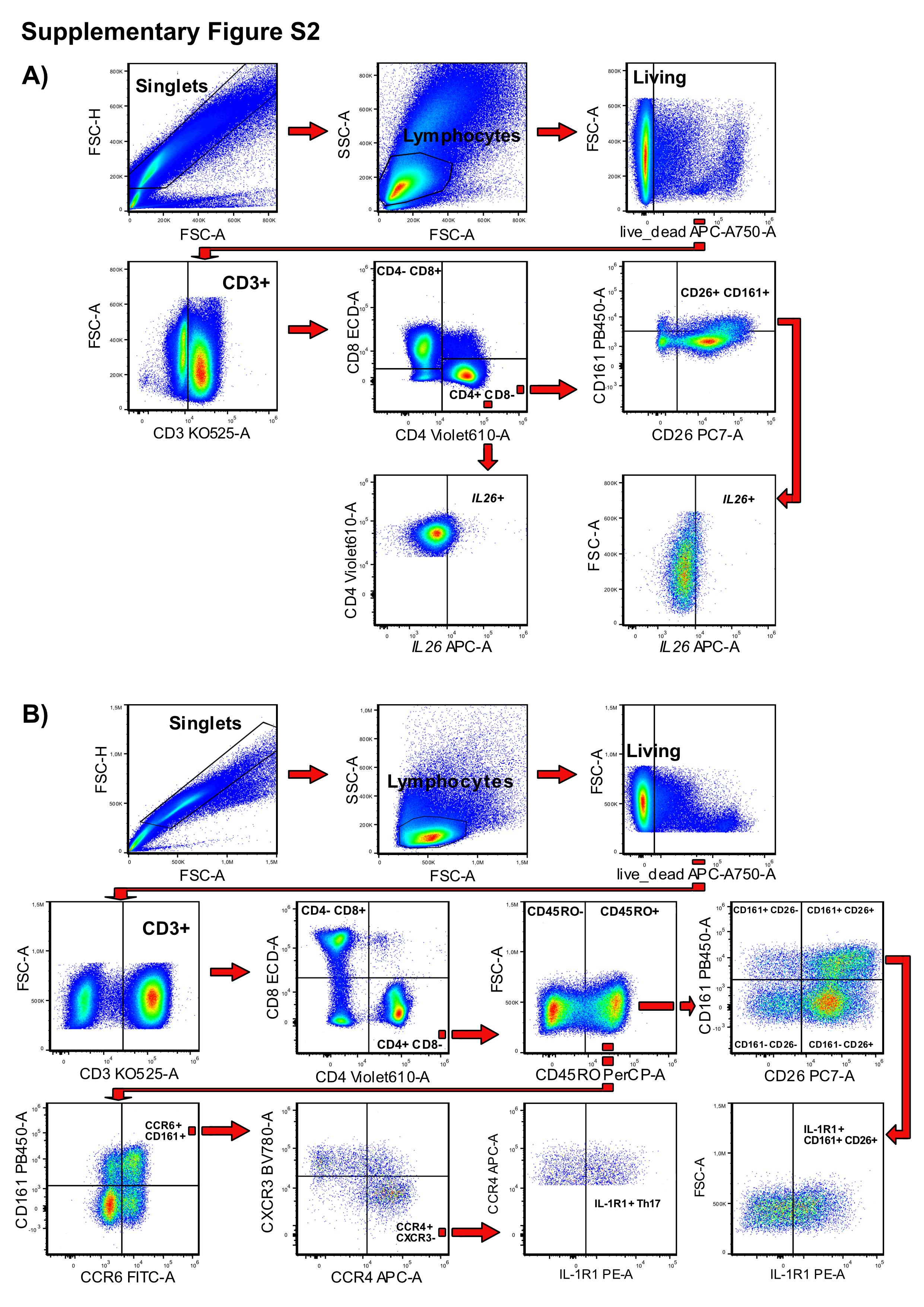

Supplement: Supplementary file 2 [file DataSheet_1.zip › Supplementary Figure 2.TIFF]

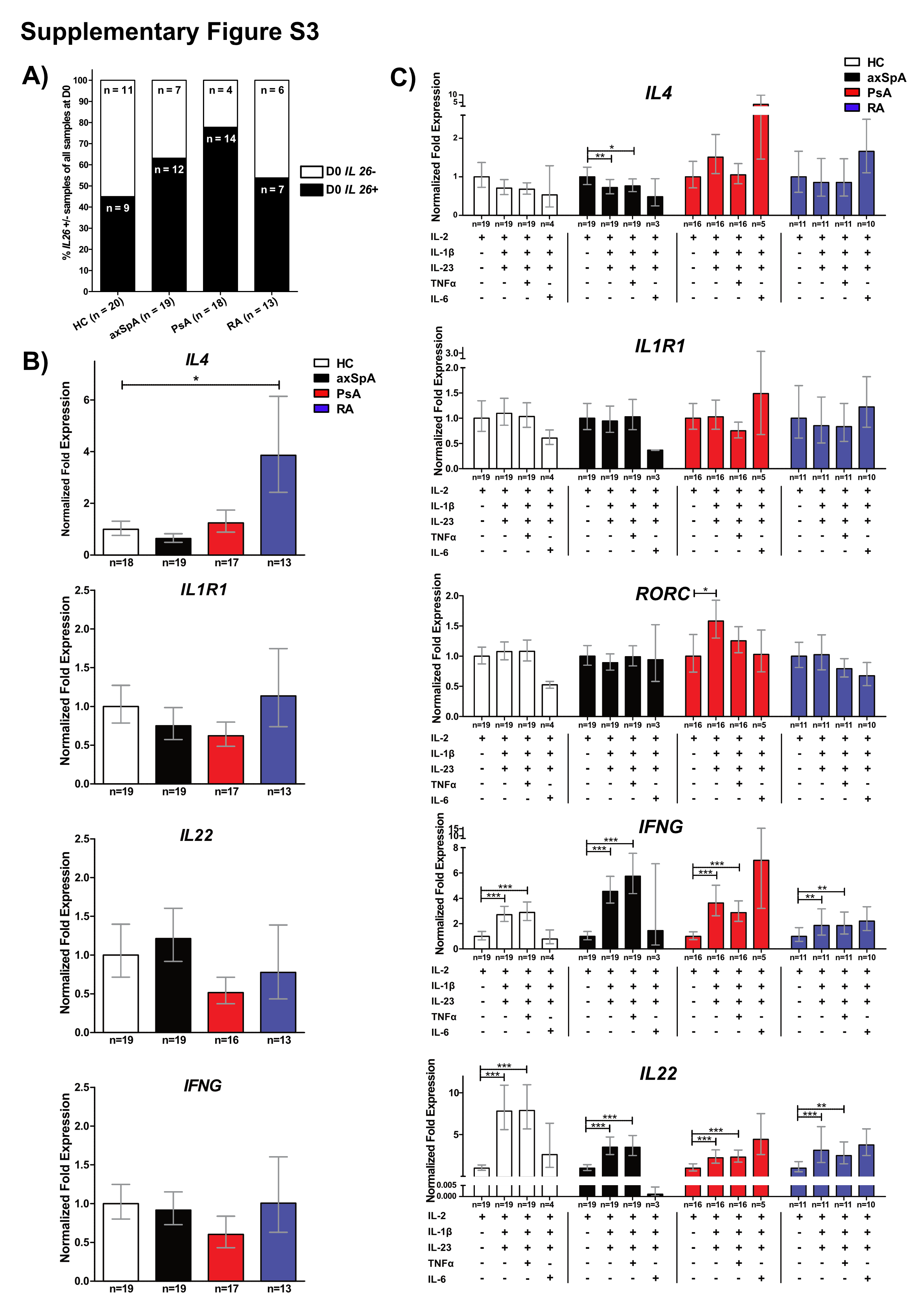

Supplement: Supplementary file 2 [file DataSheet_1.zip › Supplementary Figure 3.TIFF]

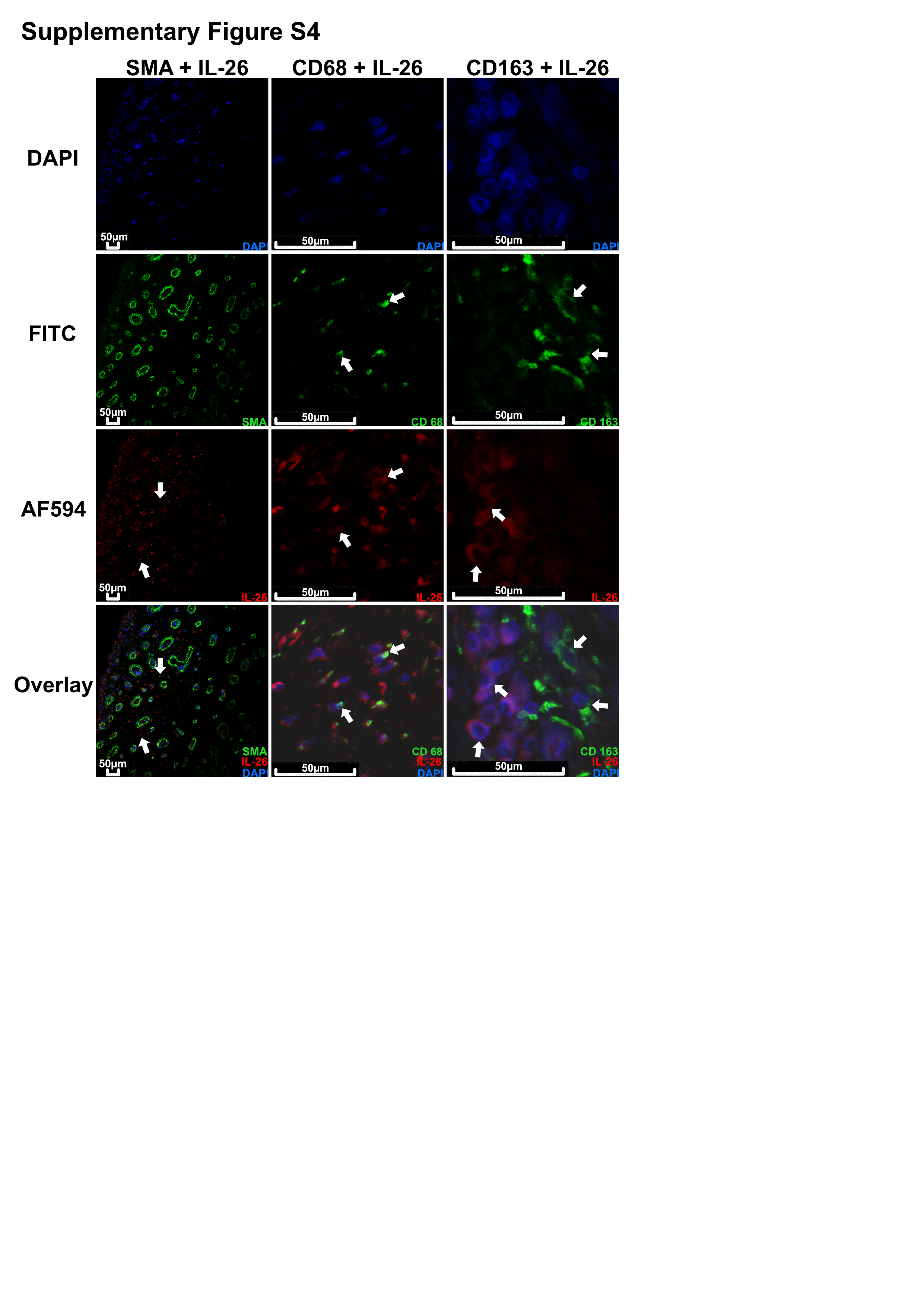

Supplement: Supplementary file 2 [file DataSheet_1.zip › Supplementary Figure 4.TIFF]
